# Adaptations in irrigated agriculture in the Mediterranean region: an overview and spatial analysis of implemented strategies

**DOI:** 10.1007/s10113-019-01494-8

**Published:** 2019-04-24

**Authors:** Kina Stientje Harmanny, Žiga Malek

**Affiliations:** Institute for Environmental Studies (IVM), Vrije Universiteit Amsterdam, De Boelelaan 1087, 1081 HV Amsterdam, The Netherlands

**Keywords:** Adaptation, Irrigation, Land management, Water management

## Abstract

In order to meet future food demand while sustainably managing available land and water resources, irrigated agriculture in semi-arid regions needs to adapt as a response to climate and socio-economic change. In this study, we focus on the Mediterranean region, a dynamic region, which is highly dependent on irrigated agriculture. We provide insight on adaptation strategies implemented on farm level, by doing a systematic review of studies in the region. Our analysis reports 286 implemented adaptations, on 124 different locations throughout the Mediterranean. Additionally, 142 drivers and 324 effects of adaptations were noted. We identified 31 adaptation strategies in 5 main categories: (1) water management, (2) sustainable resource management, (3) technological developments, (4) farm production practices, and (5) farm management. Strategies in the categories water management and farm production practices are most often implemented by farmers in the region. The main driver in the area is water scarcity and adaptations often affected water use and resources in addition to farm practices. Subsequently, we studied the spatial context of adaptations by analyzing the location factors of the five main strategies, using Geographic Information Systems and maximum entropy modeling. Our results show that farmers are more likely to adapt in less rural areas with lower poverty values and better market access, and in areas with higher temperatures and less rainfall. This demonstrates that both biophysical and socio-economic factors determine the context in which adaptations are implemented and that considerable spatial variability in the area exists.

**Electronic supplementary material:**

The online version of this article (10.1007/s10113-019-01494-8) contains supplementary material, which is available to authorized users.

## Introduction

The agricultural sector is of vital importance for the Mediterranean. In addition to ensuring food security, the sector employs a considerable share of the population and contributes significantly to the regions’ economy. Although the climatic conditions in the area are suitable for growing a wide variety of crops, irrigation is essential to maintain consistent yields (Daccache et al. 2014). With around 30% of the cropland being irrigated, it is the largest consumer of freshwater in the Mediterranean region (FAO 2016; Plan Bleu 2008). Due to high population density and semi-arid climatic conditions, the Mediterranean is among the most water-scarce regions, posing serious constraints on irrigation (Mekonnen and Hoekstra 2016; United Nations 2017). Despite the differences between countries in the region regarding water withdrawal and available water resources, in many cases, abstraction exceeds recharge (Daccache et al. 2014). Water quality degradation, caused by over extraction, often makes water resources unavailable for irrigation, forcing farmers to find new approaches to cope with water scarcity (Berahmani et al. 2012; Pisinaras et al. 2010). Moreover, additional pressures on water resources are expected due to future urbanization and demographic and economic growth (García-Ruiz et al. 2011; Malek and Verburg 2017a; Plan Bleu 2008). Simultaneously, water availability in the region is decreasing as a consequence of climate change, particularly due to rising temperatures and shifting precipitation patterns (Giorgi and Lionello 2008; Grasso and Feola 2012; Iglesias and Garrote 2015; Iglesias et al. 2011a, 2011b; IPCC 2014). It is estimated that the gross irrigation requirements will face an increase between 4 and 18% if irrigated agriculture does not adapt to these changing conditions (Fader et al. 2016).

Apart from water scarcity, other environmental factors also put pressure on farmers in the Mediterranean. Besides global socio-economic drivers, farmers experience changes on a local level such as a decreasing agricultural output due to soil erosion (Calatrava et al. 2011; Quinton et al. 2010). Intensive farming and increasing use of pesticides and fertilizers have greatly benefitted the overall farm efficiency. However, the adverse effects on soil and the environment increase the need for a more sustainable use of resources through more nature-based solutions (Keesstra et al. 2018). Furthermore, regional political instability can lead to land abandonment and agricultural migration (De Haas 2011). Due to these changing conditions, farmers increasingly struggle to maintain their livelihoods (Lorent et al. 2008; Rouabhi et al. 2016). At national and international level, policies are implemented as a response to these challenges. Irrigated agriculture is directly influenced by these policy measures. Examples include subsidized irrigation in the form of water made available at low cost to stimulate food production, water pricing, and quota on water use to limit water use (Iglesias and Garrote 2015) and promoting more sustainable use of natural resources through subsidies on organic farming (Vincent and Fleury 2015).

Mediterranean farmers have been responding and adapting to changes in their environment throughout history (Iglesias et al. 2011b; Varela-Ortega et al. 2016). Nevertheless, the conflux of current challenges require action to not only respond to existing issues but also to adapt more proactive strategies (Biagini et al. 2014) in order to meet future food demand in a sustainable way. Adaptation to climate change has so become a central part of climate research (Grothmann and Patt 2005), demonstrated also by a considerable increase in literature on adaptation in the recent years (Berrang-Ford et al. 2015). Research on adaptation has transformed into a comprehensive view that now includes both socio-economic and environmental aspects (Varela-Ortega et al. 2016). Different adaptation strategies have been reported in the Mediterranean. Examples include switching to more efficient irrigation systems as a response to water scarcity (Sese-Minguez et al. 2017) growing cover crops to control soil erosion (De Graaff et al. 2010) or switching crop types (Schilling et al. 2012). These typologies either focus on specific countries, farm type, decision-making factors of farmers, trade-offs, or specific drivers such as climate change, while others represent possible strategies rather than ones actually adopted (Biagini et al. 2014; Frija et al. 2016; Iglesias and Garrote 2015; Laoubi and Yamao 2009; Meinke et al. 2009; Schilling et al. 2012; Smit and Skinner 2002). This existing literature provides valuable insights on classifications and is the basis for defining new categories specified to the unique circumstances and characteristics of irrigated agriculture in the Mediterranean.

Despite numerous studies that focus on individual cases of adaptation in the Mediterranean and the importance of tracking and monitoring adaptation pathways to inform the decision-making process on (future) adaptations (Hermans et al. 2017), a general overview on adaptation in the region is missing. Moreover, the spatial variability and specific context of agricultural adaptations in the Mediterranean has not been analyzed. Existing categorizations of adaptations mostly contributed to the theoretical understanding of adaptations, whereas more information on strategies that are actually implemented is needed. Therefore, in this study, we aim to identify implemented farm level adaptations and their drivers and effects in Mediterranean irrigated agriculture, in addition to exploring patterns regarding frequency and distribution. We present a general overview of strategies used in the Mediterranean derived from a systematic review. Finally, we analyze the spatial context of these adaptations by investigating the influence of both biophysical and socio-economic factors.

## Methodology

### Study area

**Fig. 1:**
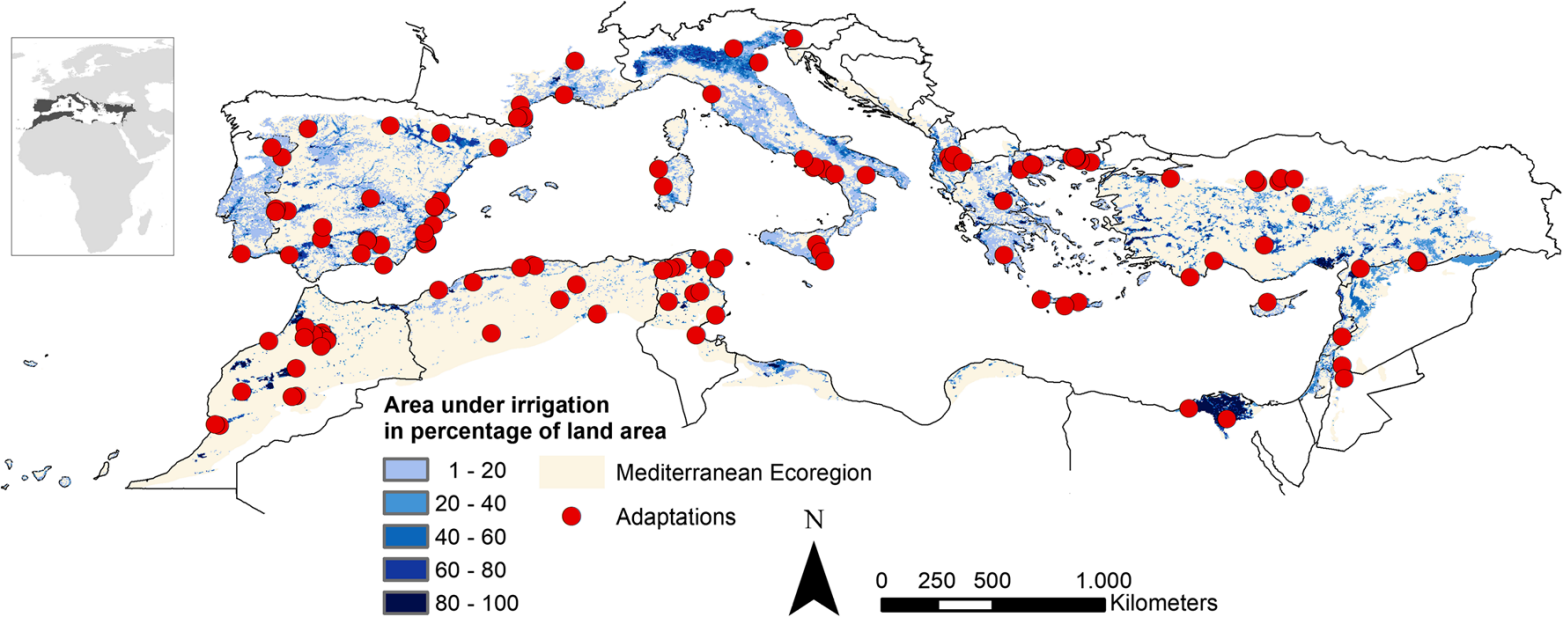
Study area: the parts of the Mediterranean Ecoregion under irrigation varying from a low to a high percentage (Olson et al. 2001; Siebert et al. 2005, 2013), additionally depicted are the 124 case locations where adaptations were reported

We focus on the Mediterranean ecoregion, which delineates the area with a typical Mediterranean climate and biogeographic conditions (Olson et al. 2001). The area is spread over 25 countries surrounding the Mediterranean sea (Fig. 1). More specifically, we performed our analysis on the areas under irrigation, using the data on areas equipped with irrigation (Siebert et al. 2005, 2013). Spread out across three continents, the region is home to approximately 420 million people of which one-third is concentrated along the shared coastline. The area shares similar climatic characteristics of long, hot, and dry summers and mild winters. At the same time, a great variety of natural and social-economic differences exists between countries, leading to variability in agricultural activities throughout the area. Currently, almost a third of the cropland is irrigated (FAO 2016) with differences in terms of intensity (Siebert et al. 2005, 2013), efficiency, and used water resources (Fader et al. 2016).

### Systematic review of case studies

We performed a systematic review to compare results from different studies and identify the various adaptation strategies reported in the study area, together with their drivers and effects. We aimed to gather studies evenly distributed across the study area, thus enabling countries for which no (recent) data was available, to still be included in the analysis. Studies were selected after using the search scope in web of science: Topic = (farm “AND” adaptation “OR” adapt “AND” [country name]) or Topic = (adaptation “OR” adapt “AND” irrigation “OR” irrigate “AND” [country name]). Timespan = 2007–2017 and Search language = English. This search resulted in 451 possibly relevant publications which were then scanned based on title and abstract after which 29 were included for analysis. Subsequently, the process was repeated in the database of Science Direct using the following search scope: Topic, title, abstract = (irrigation “OR” irrigate “AND” [country name] “AND NOT” experiment). This resulted in 431 publications of which 24 were included. We found an additional 32 studies through snowballing, bringing the total amount to 85. We included one conference proceeding for France as a limited amount of studies was available, and one study dating from 2003 for Syria as no recent data could be found. Studies were included if they reported on location (sub-national level), activity (cropland, orchard, vineyard, greenhouse, horticulture), presence of irrigation, implemented adaptation strategy, driver, effects, and crop type (optional). We excluded temporary experimental sites.

The 85 studies report on 124 farm locations (Fig. 1) describing 286 agricultural adaptations and 142 drivers. To avoid misrepresentation, only the drivers reported as most prevalent are included for analysis. The effects of adaptations are mentioned 324 times. The main methods used in the studies are case studies of specific locations and regions, either comparative (e.g. organic versus conventional farming) or evaluative (e.g., did the implementation of drip irrigation improve water use efficiency?). Many authors conducted in-depth interviews or surveys with farmers. An overview of the studies used in the analysis is provided in Online Resource 1. We reported the farm locations as described in the studies in coordinates, when no exact location was given, a point from the middle of the indicated area was taken. These case location points served as the basis for both reporting data from the systematic review as for the maximum entropy modeling needed for the spatial analysis. Therefore, one study can report on several case locations (farms) with potentially multiple adaptations, drivers, and effects.

Numerous definitions of agricultural adaptations are formulated in the literature. In this study, we define adaptations as strategies implemented on farm level as a purposeful response to a new set of risks and conditions (Bryant et al. 2000; Cooper et al. 2013) to enhance the ability to cope with external stresses (Brooks 2003). Based on the case study evidence from the systematic review combined with the existing frameworks on adaptations, five main categories of implemented strategies were identified. The term drivers is broadly interpreted since it includes both general driving forces and local pressures (Geist and Lambin 2002) and is used for all conditions and factors influencing farmers in irrigated agriculture to adapt. Effects of adaptations are defined here as a change directly resulting from an adaptation.

### Analysis of location factors on spatial probability

We investigated the effects of 19 location factors (Table 1) on the implementation of specific adaptation categories in the study area. This way, we could test the influence of biophysical and socio-economic factors on the spatial distribution of different adaptation strategies in the Mediterranean. The focus of the included location factors is on socio-economic variables, which are likely to influence future adaptations (Reidsma et al. 2010), and biophysical variables which have been demonstrated to influence the type and intensity of cropland activities, particularly irrigated cropland (Malek and Verburg 2017b). Together, these factors affect the agricultural system as a whole by influencing the adaptive capacity of farmers and the specific type of responses (Bryant et al. 2000). Furthermore, location factors are often considered as representative for drivers of change in agricultural land use (Van Vliet et al. 2015) and the geographic location of a farm has been linked to specific farm production practices such as soil management (Milgroom et al. 2007). All data used is publicly accessible global data on socio-economic and environmental characteristics. We operated on a 2-km spatial resolution.

**Table 1:** Description of the socio-economic, biophysical, and other location factors included in the analysis

Location factor	Unit/description	Resolution	Date	Source
Socio-economic				
Population density	People/km^2^	1 km	2010	CIESIN (2015)
Rural population	Rural population/km^2^	1 km	2000	CIESIN et al. (2011)
Market accessibility	Index (0–1)	1 km	2000–2010	Verburg et al. (2011)
Market influence	USD/person (ppp)	1 km	2000–2010	Verburg et al. (2011)
Accessibility	Distance to roads (m)	vector	1999	NGIA (2015)
Poverty	Share of population living in poverty	30 arc sec	2004	Elvidge et al. (2009)
Soil				
Drainage	Drainage class	1 km	2010	Hengl et al. (2014)
Sand content	Sand mass in %	1 km	2010	Stoorvogel et al. (2016)
Clay content	Clay mass in %	1 km	2013	Stoorvogel et al. (2016)
Cation exchange capacity (CEC)	cmol/kg	1 km	2010	Hengl et al. (2014)
pH	log(h+)	1 km	2010	Hengl et al. (2014)
Organic carbon content	g/kg in the top 50 cm	1 km	2013	Stoorvogel et al. (2016)
Soil depth	cm	1 km	2013	Stoorvogel et al. (2016)
Terrain				
Altitude	m above sea level	1 km	2005	Hijmans et al. (2005)
Slope	Slope degrees	1 km	2005	derived from Hijmans et al. (2005)
Climate				
Precipitation	Annual precipitation (sum of monthly means) in mm	1 km	2005	Hijmans et al. (2005)
Temperature	Temperature (mean of monthly means) Celsius degree	1 km	2005	Hijmans et al. (2005)
Solar radiation	Horizontal surface irradiation (kWh/m^2^), 1998–2011 mean	1.5 arc minute	2012	Huld et al. (2012)
Other				
Potential evapotranspiration (PET)	Annual PET in mm	1 km	2007	Zomer et al. (2008)

Six variables accounting for the influence of socio-economic factors were included for analysis. Population density and density of rural population influence, among other things, the type of activities expected in an area (Neumann et al. 2015). Market influence represents the capital available to invest in expansion or intensification in agricultural production, whereas accessibility to national and international markets indicates the potential for farmers to market their goods (Verburg et al. 2011). The influence of infrastructure was analyzed by the distance to roads, and lastly, a poverty index was included (Elvidge et al. 2009). Agricultural activities and the suitability of land for growing crops are influenced by biophysical variables (Panagos et al. 2013), which we represented by seven variables for soil characteristics: drainage class, sand, clay, organic content, cation exchange capacity (CEC), pH, and soil depth. Given that cropland is mostly situated on lower altitudes, contrary to vineyards which are more often found on slopes in the study region, the variables altitude and slope are included (Malek and Verburg 2017b). Climatic variables potentially limit the growth of crops, possibly influencing the implementation of adaptation strategies (Bryant et al. 2000; Smit and Skinner 2002). Climate variables included in the analysis are temperature, precipitation, solar radiation, and potential evapotranspiration.

To statistically analyze the influence of these variables, the coordinates of the 124 case locations were reported using a Geographic Information System (GIS). We then performed maximum entropy modeling using MaxEnt (Phillips et al. 2017). Maximum entropy is a suitable approach when studying the spatial distribution in case of presence-only data with limited information on the locations of absence of a specific phenomenon (Elith et al. 2011). Moreover, maximum entropy has been identified among most suitable methods to deal with relatively small samples (Wisz and Guisan 2009). MaxEnt identifies the most optimal distribution based on the limited information on the spatial distribution and different spatial characteristics (Phillips et al. 2006). The approach has been used to study numerous aspects of agricultural management: carbon sequestration of different land management regimes, spatial distribution of crops, and changes to the cropland extent due to climate change (Duan and Zhou 2013; Liu et al. 2015; Luedeling and Neufeldt 2012; Machovina and Feeley 2013). We performed maximum entropy modeling on the extent of irrigated cropland in the Mediterranean, which is how we eliminated areas where the studied process is unlikely to occur. Maximum entropy was modeled for all adaptation types separately and combined (only adaptation). MaxEnt provides values on how different location factors contribute to the spatial distribution, both in terms of direction and effect size. To estimate the predictive abilities of our models, we calculated the receiver operating characteristic (ROC), and its area under curve (A.U.C.) value. Before performing our analysis, we tested the correlation between all included variables (Online Resource 3). We excluded all variables with a correlation > 0.8. The aridity index (Zomer et al. 2008) which we first wanted to include in our analysis was excluded as it was highly correlated to precipitation. All other variables were kept. To test whether our limited sample of studies was subject to spatial autocorrelation (Online Resource 4), we calculated the Moran’s I statistics, a suitable measure to detect autocorrelation (Lesschen et al. 2005).

## Results

### Overview of adaptations in the Mediterranean

**Fig. 2:**
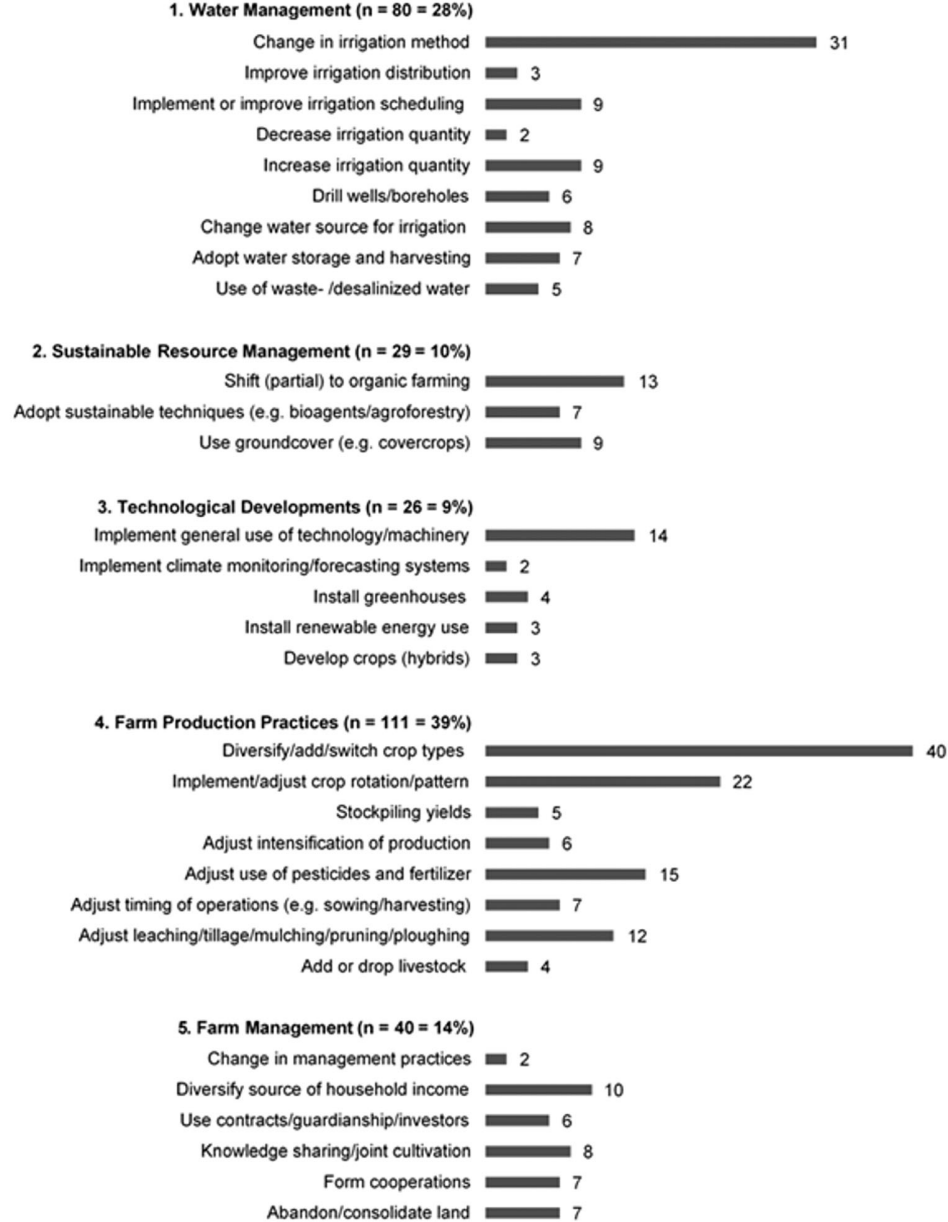
Overview of adaptation strategies currently implemented in the Mediterranean, showing 31 subcategories grouped into 5 main categories in addition to the reported number of implementations in absolute count and percentages (*N* = 286)

Based on existing frameworks and typologies of adaptations in addition to other available literature, we classified the reported 286 adaptations implemented in the Mediterranean into 31 strategies, which are grouped into 5 main categories. This resulted in an overview of reported farm level adaptation strategies as shown in Fig. 2.

#### Water management

For the scope of this study, the category water management was defined as implementing adaptation strategies directly related to the use of water for irrigation. This includes changes in irrigation methods, use of different water resources, storage, and harvesting of rainwater (Collet et al. 2015; Falloon and Betts 2010; Iglesias and Garrote 2015) but excludes strategies that potentially affect water use but also serve other purposes. Strategies within this category were the second most reported (28%) and are implemented throughout the area. Farmers often directly target changing and improving the irrigation systems by switching to more efficient irrigation methods (mostly drip) and by adjusting irrigation scheduling (Fig. 2). Improving the efficiency of irrigation was mostly reported in European Union (EU) countries of the study area. Other activities focus on changing the source of water for irrigation and water harvesting which seems especially common practice in the North-Western (NW) African countries.

#### Sustainable resource management

Strategies in this category are implemented to lower the impact of farming on the environment through more nature-based strategies (Keesstra et al. 2018). The study area is a vulnerable ecoregion facing large-scale deterioration of natural resources (Plan Bleu 2008) increasing the need for and use of these strategies. Therefore, we identified this as a separate category. Adaptations in this category were reported in 10% of the cases (Fig. 2). The most frequently used strategy is a (partial or full) shift towards organic farming, followed by using groundcover to control soil erosion or growing cover crops to attract pollinators or other insects who naturally control pests. In other cases, farmers adapted resource sustainable techniques such as implementing agroforestry or using bioagents against pests and diseases. Interestingly, adaptations from this category were only reported in EU countries.

#### Technological developments

Technological developments are frequently advocated as an adaptation in irrigated agriculture. Smit and Skinner (2002) defined this category in their overview of agricultural adaptations to climate change and included here for example weather and climate monitoring and forecasting systems. Additionally, Clements et al., (2011) discuss the many available technologies for climate change adaptation in the agricultural sector, including implementing advanced protected agriculture, using renewable energy sources or (further) mechanization of farm production practices. We excluded technology specifically aimed at managing water resources and irrigation, since this is separately categorized under water management. Adaptations in this category were reported in 9% of the cases (Fig. 2) throughout the study area. Although many options are available, only a limited amount of strategies is reportedly used in the Mediterranean. The general implementation of technology and machinery is the most frequently adopted strategy followed by implementing protected agriculture. In two cases, farmers implemented advanced weather and climate monitoring and forecasting systems. Strategies from this category are often combined with adaptations in water management, for example by installing greenhouses combined with more efficient irrigation systems (Imache et al. 2009).

#### Farm production practices

Crop management such as crop choice, rotation, and diversification are all farm production practices in addition to sowing, harvesting, mulching, and pruning. Smit and Skinner (2002) defined the category of farm production practices but included irrigation which we categorized separately. Characteristics of this category include all adjustments farmers make to existing practices, to increase the flexibility of the production process and potentially reduce exposure to risks. Adaptations in this category were the most frequently reported (39%) and are implemented all over the area (Fig. 2). Crop production diversification, by changing crop types (permanent instead of annual crops) or adding a different crop variety, is mentioned in 40 cases. This is followed by adjustments in crop rotation and pattern. In many cases, farmers simultaneously implement multiple adjustments in their farm practices. Strategies from this category are often combined with strategies from water management.

#### Farm management

The fifth category of farm management includes financial and administrative practices in addition to activities in the broader management context. This involves issues related to ownership of the farm, securing the income through insurance, shares and other investments, and knowledge building and sharing (Daoudi et al. 2013; Smit and Skinner 2002; Smith et al. 2000). Strategies in this category were reported in 14% of the cases (Fig. 2), with the most common adaptation being diversification of household income. Knowledge sharing through a participatory approach, using workshops or project management, was mainly reported in Morocco, Turkey, and Algeria, contrary to the EU where it was only reported in one case. Other methods used are management decisions concerning abandonment of remote and less productive land. Additionally, participating in voluntary land consolidation, aimed to improve the efficiency of multiple farms at once, was mentioned as an adaptation strategy (Yaslioglu et al. 2009). The distribution of these types of adaptations shows that strategies from this category are mostly “stand-alone.”

### Drivers

**Fig. 3:**
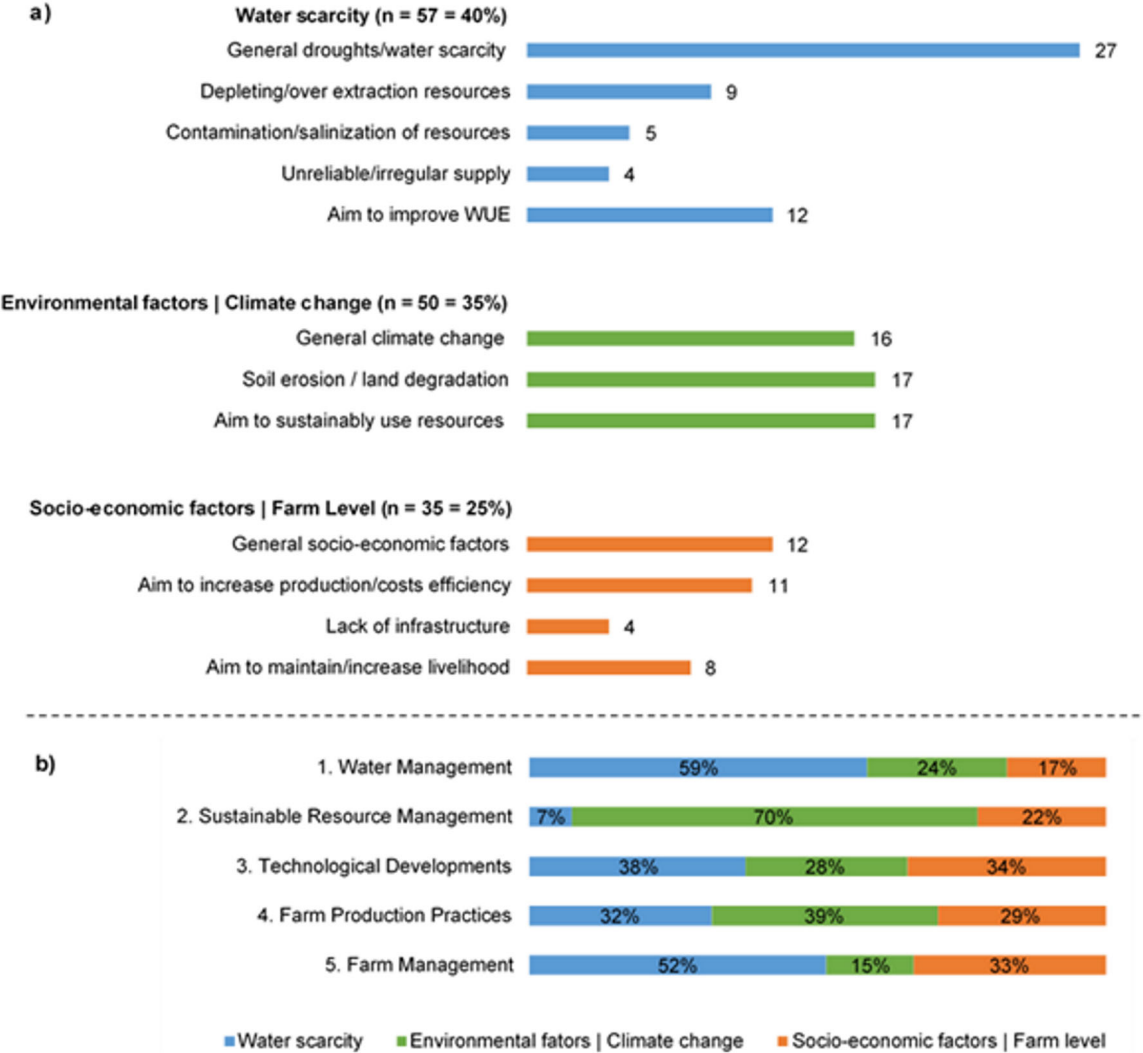
Overview of reported drivers of adaptations. **a** The 12 subcategories of drivers grouped into 3 main categories of drivers reported in absolute count and percentages (*N* = 142). **b** The relative importance of underlying drivers of each main category of adaptation strategies in percentages

A maximum of two reported drivers per case location was included in the analysis, bringing the total to 142. The drivers were subsequently classified into one of the three defined main categories. As expected, the category “water scarcity” is the predominant driver (40%) for Mediterranean farmers to adapt (Fig. 3), since water scarcity is often identified as a major threat to Mediterranean irrigated agriculture (Fader et al. 2016). Droughts and water scarcity in general are frequently mentioned. Especially in NW Africa, this is the main driver due to salinization, desertification, and unsustainable management of water resources. Farmers in Spain and Portugal are also reportedly adapting to water scarcity. Environmental factors, such as soil erosion, climate change, and the aim to sustainably manage resources, account for 35% of the drivers. Changes on a local level pressure farmers to increase the production and farm efficiency. Environmental factors and climate change are the primary drivers of sustainable resource management as explained by the relative importance (Fig. 3). Water scarcity often leads to adaptations in water and farm management. Adaptations in Greece, Turkey, and the Balkan countries are mostly driven by socio-economic changes such as the aim to increase cost efficiency or improve livelihoods.

### Effects

**Fig. 4:**
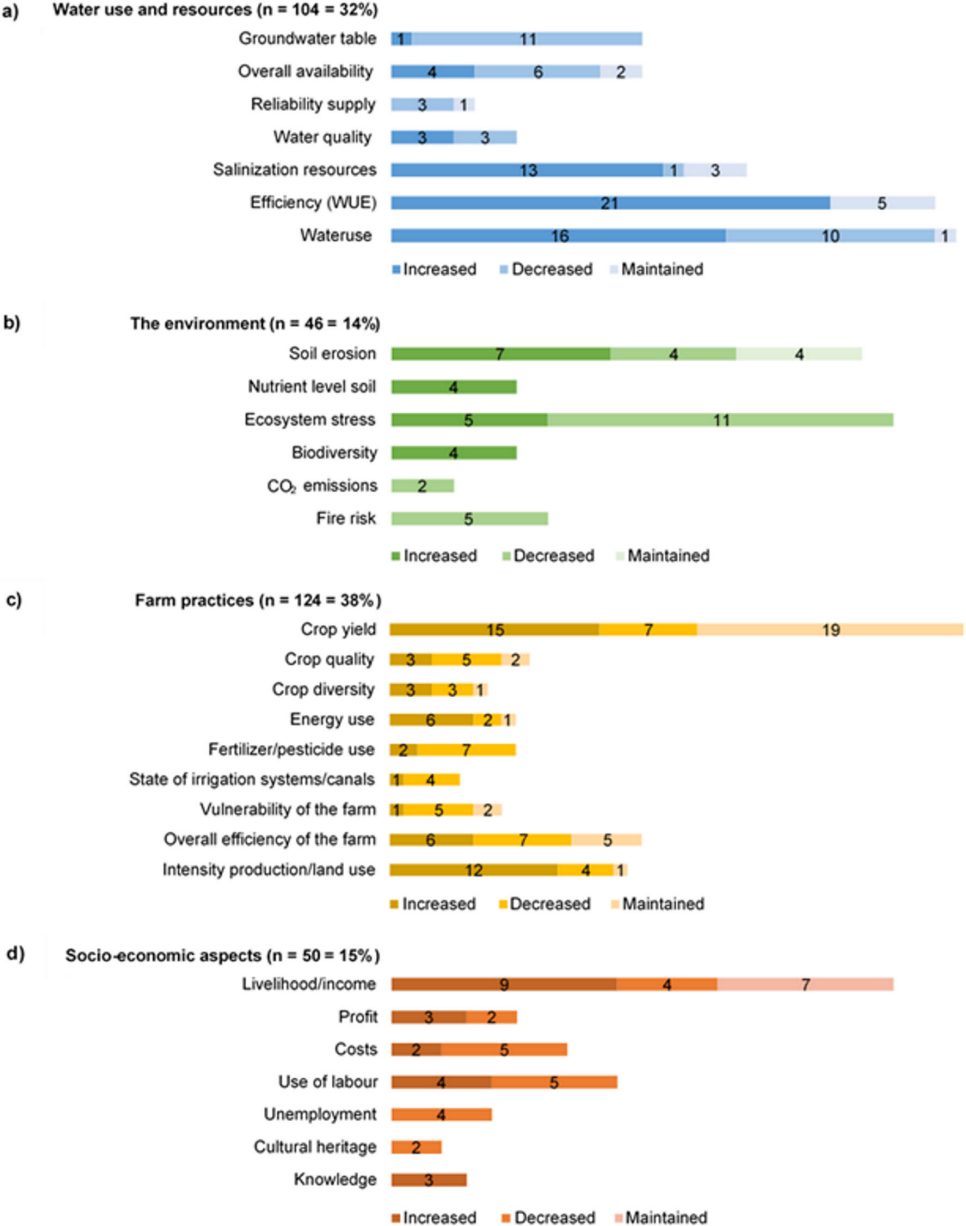
Overview of effects of implemented adaptations noted as an increased, decreased or maintained state of 29 subcategories, grouped into 4 main categories reported in absolute count and percentages (*N* = 324) of **a** water use and resources, **b** the environment, **c** farm practices, and **d** socio-economic aspects

Effects on four main categories, all containing subcategories, can be distinguished. A total of 324 effects were reported (Fig. 4) in either an increased, decreased, or maintained state. Effects on farm practices account for 38% of the total, as can be expected since adaptations in farm production practices are most prevalent. Adaptations lead to changes in the use of energy, fertilizer, and pesticides. Furthermore, this category includes the overall vulnerability and production efficiency of the farm, in addition to changes in farming intensity. We observed an overall increase in land and energy use. Crop yield fluctuates between increased, decreased, and maintained. Next in line are effects on water use and resources (32%) which includes the state of the groundwater table, surface water level, the overall availability and reliability of water supply, water use efficiency (WUE), and overall water use and salinization of resources. Overall, we see an increase in both water use and salinization of resources, known to be caused by irrigation. Additionally, the WUE increased in 20% of the cases. Effects on socio-economic aspects (15%) show an overall increase in livelihood and/or income. Profits, costs, and other aspects fluctuate between increased and decreased. The environment can be affected in multiple ways, varying from effects on soil erosion to the nutrient level in the soil. Other potential factors that are influenced include biodiversity levels, ecosystem stress (La Rosa et al. 2008), amount of CO_2_ emissions, and fire risk. An overall increase in soil erosion (though in some areas a decrease) was noted. In other cases, biodiversity levels increased and ecosystem stress decreased. Overall, the adaptations lead to numerous effects, with adaptations in water management mainly influencing water use and resources, and sustainable resource management affecting different parts of the environment. Adjustments in farm production practices strongly influence crops and farm practices in addition to other categories, whereas socio-economic effects are frequently caused by adaptations in farm management and farm production practices.

### Spatial context

Overall, we observe that farmers are more likely to adapt with favorable socio-economic conditions: in less rural areas with better market access and lower poverty levels (Table 2). At the same time, adaptation occurs more frequently distant to major roads and in areas with less developed markets. Adaptation was identified to occur on irrigated areas with less drained, deeper soil, on lower slopes and medium altitudes. In terms of climate, drier areas with higher temperatures and potential evapotranspiration (PET) are more likely to be subject to adaptation. Soil characteristics that are important for cropland activities in general, such as soil organic content, pH, and soil structure, do not play a significant role when looking at all identified adaptations.

**Table 2:** Overview of the contributions of variables to the spatial distribution for each category of adaptations in addition to the complete sample of all adaptations. The values are presented in % and describe the effect each variable has on the spatial distribution. The direction of influence is indicated as positive (+) negative (−) or unclear (±). No values mean that the variable does not contribute to the spatial distribution. The area under curve (A.U.C.) describes the predictive ability of the model

			Adaptation category									
Location factor	All adaptations		Water management		Sustainable resource management		Technological developments		Farm production practices	Farm management		
Socio-economic												
Population density	2.4	+	3.4	+	0.9	+	2.5	+	4.7	+	1.7	+
Rural population	10.1	–	8.5	–	10.6	–	0.3	+	5.4	–	4.5	–
Market accessibility	12.2	+	1.5	+	9.4	+	1.0	–	7.4	+	15.6	+
Market influence	17.0	–	4.5	+	25.4	+	10.1	–	19.1	–	29.9	–
Poverty	14.2	–	22.0	–	7.8	–	5.3	–	11.8	–	2.6	–
Road distance	4.1	+	4.8	+	0.8	+			3.8	+	1.2	+
Soil												
Drainage class	6.5	–	13.4	–	1.7	–	7.1	–	2.8	–	1.9	±
Sand content	1.1	–	0.4	–	5.3	±	1.4	±	4.9	–	10.5	–
Clay content	1.8	+	2.6	+	7.0	±	1.2	±	1.2	+		
Cation exchange capacity (CEC)			1.2	+	5.7	–						
pH	0.6	+	1.5	+	2.2	–	0.6	+	4.7	+		
Organic carbon content	0.4	–	1.0	–	4.8	–	7.6	–	1.5	–		
Soil depth	2.3	+			2.4	±			1.4	+	0.2	+
Terrain												
Altitude	3.9	±	11.0	+	4.6	–	47.7	–	2.2	+	1.9	–
Slope	2.0	–	3.2	+	5.3	+	5.0	–	3.7	+	0.9	–
Climate												
Temperature	4.5	+	7.0	+			0.2	+	5.0	+	0.1	+
Precipitation	7.9	–	8.9	–	2.5	±	1.1	–	8.2	–	0.6	+
Solar radiation	4.4	±	5.1	+	2.2	–	8.6	±	5.9	±	10.1	±
Other												
Potential evapotranspiration	4.4	+	0.1	+	1.4	–	0.3	–	6.3	+	18.0	+
A.U.C.	0.83		0.90		0.91		0.95		0.87		0.92	

**Fig. 5:**
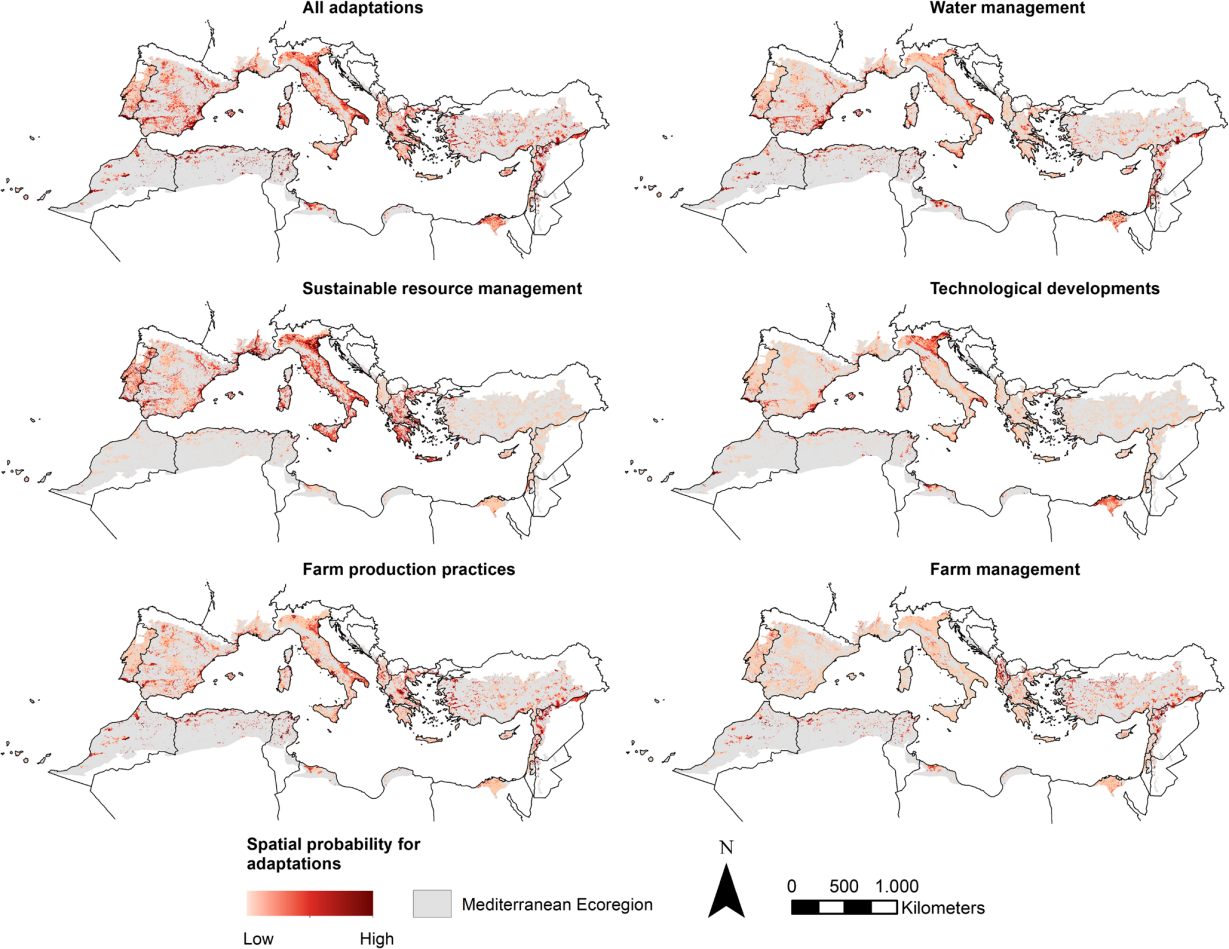
Spatial context maps, depicting the spatial variability and probability for adaptations in general and the five separate categories. High-resolution images for each spatial context map are provided in Online Resource 2

Compared to other adaptation types, water management occurs in areas with less poverty, and is considerably less affected by market accessibility and influence (Table 2). Adaptations in water management in the Mediterranean were identified to occur in areas with poorly drained soils, and higher temperatures. Moreover, we found that these are more likely to be found close to hilly and mountainous areas (higher altitude and steeper slopes). The spatial distribution (Fig. 5) clearly depicts that this type occurs in more densely populated parts and coastlines of the Mediterranean.

Sustainable resource management was identified to be more likely in areas with a lower share of rural population that are well connected to markets. Soil structure (sand and clay content), CEC, and organic content together contribute 22.8% to the spatial distribution of sustainable resource management—soil characteristics play the biggest role compared to other adaptation types. The likelihood for sustainable resource management is higher in western EU countries and Israel and is almost unlikely to occur in Northern Africa and Turkey (Fig. 5). The effect of market influence can be explained by high expected costs since this strategy often depends on subsidies (Gomez et al. 2008).

The spatial distribution of implementing technological developments is not affected by market accessibility and rural population (Table 2). The location of this type of adaptation is mostly influenced by altitude and predominantly occurs in lower elevations. Technological developments are more likely to be found on poorly drained soils with a lower organic content. The probability for technological development is higher near larger cities in the area, which generally have more evolved infrastructure, whereas the rural areas of NW Africa, the European Union, and Turkey have lower values (Fig. 5). Adaptations in farm production practices were identified in areas with a higher population density, lower poverty levels, but also lower market influence (Table 2). Climate plays an important role: less favorable climate conditions can explain 25.4% of the spatial distribution of farm production practices. Areas with a high probability for this category are more evenly distributed across the area, corresponding with the findings in the systematic review as they were reported in almost all parts of the Mediterranean (Fig. 5). However, central parts of Spain, Turkey, and Morocco, and significant areas in the Po valley and the Nile Delta also have a lower probability for this category.

The last category of farm management is particularly present in areas with better market access, but lower market influence—meaning closer to markets which are not necessarily well developed. Moreover, adaptation in farm management occurs in areas with considerably lower sand content. Similar to farm production practices, climate influences the spatial distribution of farm management. However, solar radiation and high PET alone were identified to contribute 28.1% to the spatial distribution (Table 2). This category shows high probability in the rural parts of Spain, Turkey, Northwest Africa, and the Western Balkans (Fig. 5). In this area, strategies within this category have indeed been reported in the form of joint cooperation and knowledge sharing (Cakmak et al. 2008; Lelandais 2016).

The results of the maximum entropy modeling for the five separate categories, in addition to an analysis of all case locations reporting on adaptations, are summarized in Table 2. Overall, the maximum entropy models show a good fit (described by the high A.U.C. values between 0.83 and 0.95; Table 2), demonstrating that the selected location factors can, to a certain extent, explain the spatial variation of adaptation. Spatial distribution maps demonstrate considerable spatial variability across the Mediterranean region for implementing different adaptation strategies (Fig. 5 and Online Resource 2). All probability maps presented are available on www.environmentalgeography.nl.

## Discussion

### Implications

Improving the irrigation efficiency is often advocated among the most suitable adaptation strategies to improve the sustainability of water management, particularly in the Mediterranean region (Daccache et al. 2014; Fader et al. 2016). In this study, we present, for the first time, a general overview of different adaptation strategies to improve the irrigation efficiency in the region, but also other motivations behind adaptation of irrigated cropland. Improving the irrigation efficiency is indeed frequently implemented by farmers in the region, however limited by high investment costs behind privately initiated large-scale implementation. Such large-scale implementations often included additional incentives for farmers, mostly as subsidies (Ameur et al. 2017; Özerol and Bressers 2017; Poussin et al. 2008; Soto-García et al. 2013). Moreover, we show that there is a wide variety of different types of adaptation strategies related to irrigated areas. Often, these are simplified to a single strategy, or described only by different levels of irrigation efficiency in other studies (Fader et al. 2016; Malek et al. 2018). We demonstrate that the adaptive capacity of farmers in the Mediterranean varies greatly across the region (and within countries)—uniform adaptation assumed in land use and integrated assessment models could therefore misrepresent the locations where future improvements to irrigated cropland are expected (Fader et al. 2016; Iglesias et al. 2011a, b; Malek and Verburg 2017b). Our results therefore improve our knowledge on which areas in the Mediterranean to target when implementing adaptation strategies, and the obstacles that might be limiting successful adaptation (e.g., rurality, poverty, market influence, but also unfavorable climate and soil characteristics).

Implementing sustainable resource management strategies is strongly concentrated in EU countries, which is in line with the growing demand in this area for organic products. Sustainable management is often stimulated through governmental subsidies on which these farms heavily depend for profitability (Gomez et al. 2008; Vincent and Fleury 2015). On the other hand, this category included strategies like using cover crops, which requires low investment costs and was thus expected to be more prevalent throughout the area. However, the amount of studies in the systematic review reporting on this strategy was limited, with a total of 29 cases being reported. The same applies to the category technological developments, which has been reported 26 times; nevertheless, a clear pattern of occurrence could still be observed. This presents an interesting contrast to the literature reporting on cases around the world, describing a large set of available strategies (Clements et al. 2011; Smit and Skinner 2002) and the importance of acces to technology for farmers to reduce climate-related risks (Roco et al. 2015). This discrepancy could be influenced by our classification of advanced irrigation systems into the category water management.

Contrary to the two aforementioned categories, farm production practices were frequently reported (111 times) and distributed throughout the area. This high adaptation rates can be related to the generally low costs involved in addition to the relatively easy applicability, since it is often more manageable for farmers to adjust existing practices then to invest in new ones. Other strategies in terms of “high return on investment” are adaptations in farm management. For example, knowledge sharing with and between farmers increased the efficiency of the farms involved which led to a more sustainable way of managing resources (Menegaki et al. 2007).

Many studies report on the potentially harmful effects of climate change if Mediterranean irrigated agriculture does not adapt to the changing conditions (Fader et al. 2016; Falloon and Betts 2010; Giorgi and Lionello 2008; Grasso and Feola 2012), and previous research suggest that a higher exposure to extreme climatic conditions increases adaptation rates (Reidsma et al. 2010) particularly when changes are percieved on a local level (Niles et al. 2015). However, several studies reported on farmers implementing adaptations without a direct awareness of the driver climate change behind it (Bento et al. 2009; Cohen et al. 2014; Duarte Alonso and O’Neill 2011). In one case, farmers explicitly denied the existence of climate change. Rather, they stated that their adjustments were based on their experience and observations (Cohen et al. 2014). Despite the severity of climate change and its impact on the other drivers, the results showed all three categories of drivers were (almost) equally represented in the area.

To limit impacts on water resources, adaptations in water management show great potential. However, other practices such as implementing crops with lower water needs also marked a drop in irrigation (Berahmani et al. 2012), indicating that effects on water use and resources can also be obtained by adjusting other practices. This could impact the decision-making process of farmers who aim to increase their WUE but either lack the means to invest in more efficient irrigation or have already done so. Moreover, several adaptation strategies have multiple effects at once, which is shown by the number of reported effects (324) compared to the number of reported adaptations (286).

In this study, we present a novel approach on modeling maximum entropy to identify the spatial context of adaptations. There are numerous environmental studies that studied the spatial determinants behind landscape configurations or land change processes. Examples range from identifying the spatial distribution of land management types (Malek and Verburg 2017b), spatial drivers of wetland conversion (Van Asselen et al. 2013), future land system change (Van Asselen and Verburg 2013), and reconstructing historic land change (Fuchs et al. 2013). However, using a combination of a systematic review and spatial analysis has not yet been applied to study adaptation, or the spatial context of decision-making in general on such a large scale. Mapping and modeling adaptation is difficult, due to the complex nature of decision-making behind adaptation (Holman et al. 2019). Nevertheless, our models show a good fit, thereby demonstrating that this method can also be used to identify spatial variability and context in which adaptations are implemented.

### Discussion of the methodology

A systematic review is a suitable approach to study adaptation, and it has been applied on other land change-related processes to study the drivers and effects of changes to land use and land cover, and land management. Examples range from agricultural land use change (Van Vliet et al. 2015), wetland conversion (Van Asselen et al. 2013), deforestation (Geist and Lambin 2002), urban expansion (Seto et al. 2011), and agricultural intensification in the tropics (Keys and McConnell 2005). Often, these approaches are global or cover a larger region (e.g., biome), without providing knowledge on the representativeness of the studies for a specific region (Magliocca et al. 2015). Our study focuses on a more homogeneous region, increasing the transferability of our results to other areas in the Mediterranean region.

One of the main limitations of our systematic review is the fact that our analysis is based on documented adaptation which was limited for some countries due to studies only published in languages other than English resulting in exclusion from the analysis. Nevertheless, we tried to avoid the bias towards more developed countries in the region by specifically looking for studies by country name. As a result, our studies are distributed across the whole Mediterranean region. While we reduced the potential correlation among the included variables (Online resource 3), we did identify spatial autocorrelation to a certain extent (Online resource 4). Documented adaptation studies tend to cluster in terms of market accessibility and influence, altitude, PET, and slightly clustered in terms of soil variables, slope, climate and road distance. On one side, this suggests that some of the areas tend to be studied more (e.g., Spain) and some remain understudied (e.g., Middle East and Turkey). On the other hand, this could also be explained as higher likelihood for adaptation in specific areas, or areas sharing the variables identified as spatially autocorrelated. Additionally, a publication bias towards studies focusing on climate change adaptation possibly influenced the analysis.

The possibility to track and monitor adaptations in general is limited due to its complex nature and lack of standardization (Ford et al. 2013) and hard classification criteria for inclusion in a certain category of adaptation strategies proved challenging at times. Considering that the terminology in the literature used is sometimes ambiguous and due to the variation of available strategies, formulating categories that are not mutually exclusive was unavoidable. Broad classes used in categorizations in review studies have however been observed in other studies as well (Van Vliet et al. 2015). The same goes for the drivers affecting adaptation. In view of their complex relations and nature, their classification possibly resulted in a misrepresentation of their distribution within the three defined main categories. For example, when a study reported on a farmer adapting to lower crop yield, we classified this under the driver category “farm level.” However, the lower crop yield could very well be a result from decreasing water availability for irrigation. This becomes clear when we look at the specific drivers behind the five categories of adaptations, as the driver water scarcity only accounted for 7% of adaptations in the category sustainable resource management. Additionally, some overlap between the categories existed, as they are all interrelated and influencing each other, adding in on the complexity of separately classifying them.

The literature reports on many additional factors influencing the process of implementing adaptation strategies, including government policy (either as a driver or an adaptation). However, this study only considers farm-level adaptations, drivers, and effects and their spatial context based on the evidence retrieved from the systematic review, thereby excluding (inter)national policy as a driver. Moreover, a complete overview of the policies influencing farmers could not be retrieved from the studies used in the systematic review as not all studies reported consistently on the influence or presence of policy. Instead, we focused on the drivers influencing farmers directly—as policy is often a response to these same drivers, e.g., policies target issues concerning water, climate, or sustainable resource management. Nevertheless, adaptation to climate change is becoming a main part of national governments’ strategies (Hanger et al. 2013).

Our spatial analysis could also be subject to uncertainties, related to variables used, and locations of adaptation. Water scarcity was identified as a prevalent driver for adaptation in the Mediterranean. Although temperature and precipitation can, to a certain extent, be used as proxies for water scarcity including data on water scarcity would have improved our maximum entropy model. Such data is however unavailable on a suitable spatial resolution. To represent the adaptation locations in the study area, point data was used to represent each location. However, some studies reported on large-scale farms (of 5000 ha or more) implementing adaptations (whereas most do not). The actual extent of areas with high probability for adaptations could however be affected by the size of the irrigated area subject to adaptation.

## Conclusions

This study provides additional insights on implemented adaptation strategies in the Mediterranean area in the context of their drivers, effects, and location. Overall, the data derived from the systematic review provides useful information on the nature and dynamics of the implemented adaptation strategies on farm level. They show different effects on water use and resources, the environment, farm practices, and socio-economic aspects. The classification of adaptation strategies presented in this study is specific for the Mediterranean and is an addition to the already existing frameworks on adaptations. Moreover, we attempted to provide an overview contributing to the understanding of different adaptation strategies used in Mediterranean irrigated cropland areas.

Studying the significance of different location factors, the spatial distribution of adaptation demonstrates which areas in the region are most (or least) likely to adapt. Furthermore, we showed that the methods applied can be used to explain the spatial probability of adaptations. However, the availability of specific data on water scarcity and more documented cases of farm-level adaptation could have improved our results.

Adaptation of activities on irrigated cropland on a certain location depends on the combination of location factors, drivers, desired effects, and available strategies. We have shown that the level of rural population and poverty significantly affects the likelihood to adapt. This suggests that financial and institutional means helping the farmers to adapt are likely to be inaccessible to farmers in poorer and more rural areas. Future policies concerning adaptation of irrigated cropland in the Mediterranean should therefore target these areas.

## Electronic supplementary material

Online Resource 1 (PDF 537 kb)

Online Resource 2 (PDF 1956 kb)

Online Resource 3 (PDF 152 kb)

Online Resource 4 (PDF 146 kb)
