# Supplementary material for: Adaptations in irrigated agriculture in the Mediterranean region: an overview and spatial analysis of implemented strategies
Source: Reg Environ Change. 2019 Apr 24;19(5):1401–16. doi: 10.1007/s10113-019-01494-8 (PMC6531414; doi:10.1007/s10113-019-01494-8)
Supplement: Supplementary file 1 — (PDF 537 kb) [file 10113_2019_1494_MOESM1_ESM.pdf]

Online Resource 1 Overview of studies used in the systematic review

Overview of all the studies included in the systematic review sorted by country (listed in alphabetical order). Some studies are listed multiple times because they reported adaptations on locations in more than one country.

Albania

Abdesselam, S., Halitim, A., Jan, A., Trolard, F., & Bourrié, G. (2013). Anthropogenic contamination of groundwater with nitrate in arid region: case study of southern Hodna (Algeria). Environmental Earth Sciences, 70(5), 2129–2141. https://doi.org/10.1007/s12665-012-1834-5

Algeria

Bouarfa, S., Marlet, S., Douaoui, A., Hartani, T., Mekki, I., Ghazouani, W., Aissa, I., Vincent, B. Hassani, F., Kuper, M. (2009). Salinity patterns in irrigation systems, a threat to be demystified, a constraint to be managed: Field evidence from Algeria and Tunisia. Irrigation and Drainage, 58(S3), S273–S284. https://doi.org/10.1002/ird.524

Daoudi, A., Terranti, S., Hammouda, R. F., & Bedrani, S. (2013). Adaptation à la sécheresse en steppe algérienne : le cas des stratégies productives des agropasteurs de Hadj Mechri. Cahiers Agricultures, (4), 303–310. https://doi.org/10.1684/agr.2013.0629

Hamamouche, M. F., Kuper, M., Riaux, J., & Leduc, C. (2017). Conjunctive use of surface and ground water resources in a community-managed irrigation system — The case of the Sidi Okba palm grove in the Algerian Sahara. Agricultural Water Management, 193, 116–130. https://doi.org/10.1016/j.agwat.2017.08.005

Imache, A., Bouarfa, S., Kuper, M., Hartani, T., & Dionnet, M. (2009). Integrating “invisible” farmers in a regional debate on water productivity: The case of informal water and land markets in the Algerian Mitidja plain. Irrigation and Drainage, 58(S3), S264–S272. https://doi.org/10.1002/ird.523

Laoubi, K., & Yamao, M. (2009). A typology of irrigated farms as a tool for sustainable agricultural development in irrigation schemes: The case of the East Mitidja scheme, Algeria. International Journal of Social Economics, 36(8), 813–831.

Maachou, H. M., & Otmane, T. (2016). L’agriculture périurbaine à Oran (Algérie): diversification et stratégies d’adaptation. Cahiers Agricultures, 25(2), 25002. https://doi.org/10.1051/cagri/2016011

Rouabhi, A., Mekhlouf, A., Mokhneche, S., & Elkolli, N. (2016). Farming transitions under Socio-economic and climatic constraints in the southern part of Sétif, Algeria. Journal of Agriculture and Environment for International Development, 110(1), 139–153.

Sikor, T., Müller, D., & Stahl, J. (2009). Land Fragmentation and Cropland Abandonment in Albania: Implications for the Roles of State and Community in Post-Socialist Land Consolidation. World Development, 37(8), 1411–1423. https://doi.org/10.1016/j.worlddev.2008.08.013

Cyprus

Phillips Agboola, O., & Egelioglu, F. (2012). Water scarcity in North Cyprus and solar desalination research: a review. Desalination and Water Treatment, 43(1–3), 29–42. https://doi.org/10.1080/19443994.2012.672195

Egypt

El-Agha, D. E., Molden, D. J., & Ghanem, A. M. (2011). Performance assessment of irrigation water management in old lands of the Nile delta of Egypt. Irrigation and Drainage Systems, 25(4), 215–236. https://doi.org/10.1007/s10795-011-9116-z

Mostafa, H., & Fujimoto, N. (2015). Monitoring and evaluation of irrigation management projects in Egypt. Japan Agricultural Research Quarterly, 49(2), 111–118.

France

Bento, S., Fatima, D., Errahj, M., Faysse, N., Garin, P., Audrey, R. F., Rinaudo, J.D., Rollin, D., Schmidt, L., Varanda, M. (2009). Farmers’ relations to climate variabilities and changes: the case of groundwater users of coastal aquifers in France, Portugal and Morocco. In 9th Conference of the European Sociological Association.

Coll, P., Le Cadre, E., Blanchart, E., Hinsinger, P., & Villenave, C. (2011). Organic viticulture and soil quality: A long-term study in Southern France. Applied Soil Ecology. https://doi.org/10.1016/j.apsoil.2011.07.013

Delmotte, S., Tittonell, P., Mouret, J.-C., Hammond, R., & Lopez-Ridaura, S. (2011). On farm assessment of rice yield variability and productivity gaps between organic and conventional cropping systems under Mediterranean climate. European Journal of Agronomy, 35(4), 223–236. https://doi.org/10.1016/j.eja.2011.06.006

Vincent, A., & Fleury, P. (2015). Development of organic farming for the protection of water quality: Local projects in France and their policy implications. Land Use Policy, 43, 197–206. https://doi.org/10.1016/j.landusepol.2014.10.020

Greece

Barbayiannis, N., Panayotopoulos, K., Psaltopoulos, D., & Skuras, D. (2011). The influence of policy on soil conservation: A case study from Greece. Land Degradation & Development, 22(1), 47–57. https://doi.org/10.1002/ldr.1053

Foteinis, S., & Chatzisymeon, E. (2016). Life cycle assessment of organic versus conventional agriculture. A case study of lettuce cultivation in Greece. Journal of Cleaner Production, 112, 2462–2471. https://doi.org/10.1016/j.jclepro.2015.09.075

Gomez, J. A., Amato, M., Celano, G., & Koubouris, G. C. (2008). Organic olive orchards on sloping land: More than a specialty niche production system? Journal of Environmental Management, 89(2), 99–109. https://doi.org/10.1016/j.jenvman.2007.04.025

Karelakis, C., & Tsantopoulos, G. (2017). Changing land use to alternative crops: A rural landholder's perspective. Land Use Policy, 63, 30–37. https://doi.org/10.1016/j.landusepol.2016.12.009

Lorent, H., Evangelou, C., Stellmes, M., Hill, J., Papanastasis, V., Tsiourlis, G., Roeder, A., Lambin, E. F. (2008). Land degradation and economic conditions of agricultural households in a marginal region of northern Greece. Global and Planetary Change, 64(3–4), 198–209. https://doi.org/10.1016/j.gloplacha.2008.05.005

Menegaki, A. N., Hanley, N., & Tsagarakis, K. P. (2007). The social acceptability and valuation of recycled water in Crete: A study of consumers' and farmers' attitudes. Ecological Economics, 62(1), 7–18. https://doi.org/10.1016/j.ecolecon.2007.01.008

Panagea, I. S., Daliakopoulos, I. N., Tsanis, I. K., & Schwilch, G. (2016). Evaluation of promising technologies for soil salinity amelioration in Timpaki (Crete): a participatory approach. Solid Earth, 7(1), 177–190. https://doi.org/10.5194/se-7-177-2016

Petropoulou, E. A. (2007). Indigenous resource management and environmental degradation: southern Greece. Management of Environmental Quality: An International Journal, 18(2), 152–166. https://doi.org/10.1108/14777830710725821

Pisinaras, V., Tsihrintzis, V. A., Petalas, C., & Ouzounis, K. (2010). Soil salinization in the agricultural lands of Rhodope District, north-eastern Greece. Environmental Monitoring and Assessment, 166(1–4), 79–94. https://doi.org/10.1007/s10661-009-0986-6

Stamatis, G., Parpodis, K., Filintas, A., & Zagana, E. (2011). Groundwater quality, nitrate pollution and irrigation environmental management in the Neogene sediments of an agricultural region in central Thessaly (Greece). Environmental Earth Sciences, 64(4), 1081–1105. https://doi.org/10.1007/s12665-011-0926-y

Italy

Bojovic, D., Bonzanigo, L., Giupponi, C., & Maziotis, A. (2015). Online participation in climate change adaptation: A case study of agricultural adaptation measures in Northern Italy. Journal of Environmental Management, 157, 8–19. https://doi.org/10.1016/j.jenvman.2015.04.001

Dono, G., Cortignani, R., Doro, L., Giraldo, L., Ledda, L., Pasqui, M., & Roggero, P. P. (2013). Adapting to uncertainty associated with short-term climate variability changes in irrigated Mediterranean farming systems. Agricultural Systems, 117, 1–12. https://doi.org/10.1016/j.agsy.2013.01.005

Gomez, J. A., Amato, M., Celano, G., & Koubouris, G. C. (2008). Organic olive orchards on sloping land: More than a specialty niche production system? Journal of Environmental Management, 89(2), 99–109. https://doi.org/10.1016/j.jenvman.2007.04.025

La Rosa, A. D., Siracusa, G., & Cavallaro, R. (2008). Emergency evaluation of Sicilian red orange production. A comparison between organic and conventional farming. Journal of Cleaner Production, 16(17), 1907–1914. https://doi.org/10.1016/j.jclepro.2008.01.003

Morra, L., Cerrato, D., Bilotto, M., & Baiano, S. (2016). Introduction of Sorghum (Sorghum bicolor (L.) Moench) green manure in rotations of head salads and baby leaf crops under greenhouse. Italian Journal of Agronomy, 11. https://doi.org/10.4081/ija.2016.753

Nguyen, T. P. L., Seddaiu, G., Virdis, S. G. P., Tidore, C., Pasqui, M., & Roggero, P. P. (2016). Perceiving to learn or learning to perceive? Understanding farmers' perceptions and adaptation to climate uncertainties. Agricultural Systems, 143, 205–216. https://doi.org/10.1016/j.agsy.2016.01.001

Pezzuolo, A., Dumont, B., Sartori, L., Marinello, F., De Antoni Migliorati, M., & Basso, B. (2017). Evaluating the impact of soil conservation measures on soil organic carbon at the farm scale. Computers and Electronics in Agriculture, 135, 175–182. https://doi.org/10.1016/j.compag.2017.02.004

Reedy, D., Savo, V., & McClatchey, W. (2014). Traditional Climatic Knowledge: Orchardists' perceptions of and adaptation to climate change in the Campania region (Southern Italy). Plant Biosystems - An International Journal Dealing with All Aspects of PlantBiology, 148(4), 699–712. https://doi.org/10.1080/11263504.2013.793753

Vieri, M., & Sarri, D. (2010). Criteria for introducing mechanical harvesting of oil olives: results of a five-year project in Central Italy. Advances in Horticultural Science, 24(1), 78–90.

Jordan

Carr, G., Potter, R. B., & Nortcliff, S. (2011). Water reuse for irrigation in Jordan: Perceptions of water quality among farmers. Agricultural Water Management, 98(5), 847–854. https://doi.org/10.1016/j.agwat.2010.12.011

Lebanon

Darwish, T., Atallah, T., Francis, R., Saab, C., Jomaa, I., Shaaban, A., Sakka, H., Zdruli, P. (2011). Observations on soil and groundwater contamination with nitrate: A case study from Lebanon-East Mediterranean. Agricultural Water Management, 99(1), 74–84. https://doi.org/10.1016/j.agwat.2011.07.016

Morocco

Ameur, F., Kuper, M., Lejars, C., & Dugué, P. (2017). Prosper, survive or exit: Contrasted fortunes of farmers in the groundwater economy in the Saiss plain (Morocco). Agricultural Water Management, 191, 207–217. https://doi.org/10.1016/j.agwat.2017.06.014

Benouniche, M., Kuper, M., Hammani, A., & Boesveld, H. (2014). Making the user visible: analysing irrigation practices and farmers' logic to explain actual drip irrigation performance. Irrigation Science, 32(6), 405–420. https://doi.org/10.1007/s00271-014-0438-0

Berahmani, A., Faysse, N., Errahj, M., & Gafsi, M. (2012). Chasing water: diverging farmers' strategies to cope with the groundwater crisis in the coastal Chaouia region in Morocco. Irrigation and Drainage, 61(5), 673–681. https://doi.org/10.1002/ird.1673

Codron, J. M., Adanacioğlu, H., Aubert, M., Bouhsina, Z., El Mekki, A. A., Rousset, S., Tonzanli, S., Yercan, M. (2014). The role of market forces and food safety institutions in the adoption of sustainable farming practices: The case of the fresh tomato export sector in Morocco and Turkey. Food Policy, 49, 268-280.

Jobbins, G., Kalpakian, J., Chriyaa, A., Legrouri, A., & El Mzouri, E. H. (2015). To what end? Drip irrigation and the water–energy–food nexus in Morocco. International Journal of Water Resources Development, 31(3),393–406. https://doi.org/10.1080/07900627.2015.1020146

Kuhn, A., Heidecke, C., Roth, A., Goldback, H., Burkhardt, J., Linstädter, A., Kemmerling, B., Gaiser, T. (2010). Importance of resource management for livelihood security under Climate Change in Southern Morocco (pp. 518–543). Springer.

Kuper, M., Hammani, A., Chohin, A., Garin, P., & Saaf, M. (2012). When groundwater takes over: Linking 40 years of agricultural and groundwater dynamics in a large-scale irrigation scheme in morocco. Irrigation and Drainage, 61(SUPPL.1), 45–53. https://doi.org/10.1002/ird.1653

Lybbert, T. J., Kusunose, Y., Magnan, N., & Fadlaoui, A. (2009). Drought risk and drought response in Morocco: vulnerability, risk perceptions and drought coping among rainfed cereal farmers.

Malki, M., Bouchaou, L., Hirich, A., Ait Brahim, Y., & Choukr-Allah, R. (2017). Impact of agricultural practices on groundwater quality in intensive irrigated area of Chtouka-Massa, Morocco. Science of The Total Environment, 574, 760–770. https://doi.org/10.1016/j.scitotenv.2016.09.145

Schilling, J., Freier, K. P., Hertig, E., & Scheffran, J. (2012). Climate change, vulnerability and adaptation in North Africa with focus on Morocco. Agriculture, Ecosystems & Environment, 156, 12–26. https://doi.org/10.1016/j.agee.2012.04.021

Van der Kooij, S., Zwarteveen, M., & Kuper, M. (2015). The material of the social: the mutual shaping of institutions by irrigation technology and society in Seguia Khrichfa, Morocco. International Journal of the Commons, 9(1), 129. https://doi.org/10.18352/ijc.539

Portugal

Antunes, P., Karadzic, V., Santos, R., Beça, P., & Osann, A. (2011). Participatory multi-criteria analysis of irrigation management alternatives: the case of the Caia irrigation district, Portugal. International Journal of Agricultural Sustainability, 9(2), 334–349.

Bento, S., Fatima, D., Errahj, M., Faysse, N., Garin, P., Audrey, R. F., Rinaudo, J.D., Rollin, D., Schmidt, L., Varanda, M. (2009). Farmers' relations to climate variabilities and changes: the case of groundwater users of coastal aquifers in France, Portugal and Morocco. In 9th Conference of the European Sociological Association.

De Graaff, J., Duarte, F., Fleskens, L., & de Figueiredo, T. (2010). The future of olive groves on sloping land and ex-ante assessment of cross compliance for erosion control. Land Use Policy, 27(1), 33–41. https://doi.org/10.1016/j.landusepol.2008.02.006

Gomez, J. A., Amato, M., Celano, G., & Koubouris, G. C. (2008). Organic olive orchards on sloping land: More than a specialty niche production system? Journal of Environmental Management, 89(2), 99–109. https://doi.org/10.1016/j.jenvman.2007.04.025

Slovenia

Sušnik, A., Matajc, I., & Kodric, I. (2007). Agrometeorological support of fruit production: application in SW Slovenia. Meteorological Applications, 13(S1), 81. https://doi.org/10.1017/S1350482706002581

Spain

Alcon, F., de Miguel, M. D., & Burton, M. (2011). Duration analysis of adoption of drip irrigation technology in south-eastern Spain. Technological Forecasting and Social Change, 78(6), 991–1001. https://doi.org/10.1016/j.techfore.2011.02.001

Calatrava, J., Barberá, G. G., & Castillo, V. M. (2011). Farming practices and policy measures for agricultural soil conservation in semi-arid Mediterranean areas: The case of the Guadalentín basin in southeast Spain. Land Degradation & Development, 22(1), 58–69. https://doi.org/10.1002/ldr.1013

Cohen, M., Ronchail, J., Alonso-Roldán, M., Morcel, C., Angles, S., Araque-Jimenez, E., & Labat, D. (2014). Adaptability of Mediterranean Agricultural Systems to Climate Change. The Example of the Sierra Mágina Olive-Growing Region (Andalusia, Spain). Part I: Past and Present. Weather, Climate, and Society, 6(3), 380–398. https://doi.org/10.1175/WCAS-D-12-00043.1

Díaz, J. A. R., Urrestarazu, L. P., Poyato, E. C., & Montesinos, P. (2012). Modernizing Water Distribution Networks: Lessons from the Bembézar MD irrigation district, Spain. Outlook on Agriculture, 41(4), 229–236. https://doi.org/10.5367/oa.2012.0105

Duarte Alonso, A., & O'Neill, M. A. (2011). Climate change from the perspective of Spanish wine growers: a three-region study. British Food Journal, 113(2), 205–221. https://doi.org/10.1108/00070701111105303

Egea, F. J., Torrente, R. G., & Aguilar, A. (2017). An efficient agro-industrial complex in Almería (Spain): Towards an integrated and sustainable bio economy model. New Biotechnology. https://doi.org/10.1016/j.nbt.2017.06.009

Fagúndez, J., Olea, P. P., Tejedo, P., Mateo-Tomás, P., & Gómez, D. (2016). Irrigation and Maize Cultivation Erode Plant Diversity Within Crops in Mediterranean Dry Cereal Agro-Ecosystems. Environmental Management, 58(1), 164–174. https://doi.org/10.1007/s00267-016-0691-5

García-Prats, A., & Guillem-Picó, S. (2016). Adaptation of pressurized irrigation networks to new strategies of irrigation management: Energy implications of low discharge and pulsed irrigation. Agricultural Water Management, 169, 52–60. https://doi.org/10.1016/j.agwat.2016.02.023

González Perea, R., Fernández García, I., Martin Arroyo, M., Rodríguez Díaz, J. A., Camacho Poyato, E., & Montesinos, P. (2017). Multiplatform application for precision irrigation scheduling in strawberries. Agricultural Water Management, 183, 194–201. https://doi.org/10.1016/j.agwat.2016.07.017

Guzmán, G. I., & Alonso, A. M. (2008). A comparison of energy use in conventional and organic olive oil production in Spain. Agricultural Systems, 98(3), 167–176. https://doi.org/10.1016/j.agsy.2008.06.004

Lecina, S., Isidoro, D., Playán, E., & Aragüés, R. (2010). Irrigation modernization and water conservation in Spain: The case of Riegos del Alto Aragón. Agricultural Water Management, 97(10), 1663–1675. https://doi.org/10.1016/j.agwat.2010.05.023

Milgroom, J., Auxiliadora Soriano, M., Garrido, J. M., Gómez, J. A., & Fereres, E. (2007). The influence of a shift from conventional to organic olive farming on soil management and erosion risk in southern Spain. Renewable Agriculture and Food Systems, 22(1), 1–10. https://doi.org/10.1017/S1742170507001500

Sese-Minguez, S., Boesveld, H., Asins-Velis, S., van der Kooij, S., & Maroulis, J. (2017). Transformations Accompanying a Shift from Surface to Drip Irrigation in the Canyoles Watershed, Valencia, Spain. Water Alternatives, 10(1), 81.

Soto-García, M., Martínez-Alvarez, V., García-Bastida, P. A., Alcon, F., & Martin-Gorriz, B. (2013). Effect of water scarcity and modernisation on the performance of irrigation districts in south-eastern Spain. Agricultural Water Management, 124, 11–19. https://doi.org/10.1016/j.agwat.2013.03.019

Urquijo, J., & De Stefano, L. (2016). Perception of Drought and Local Responses by Farmers: A Perspective from the Jucar River Basin, Spain. Water Resources Management, 30(2), 577–591. https://doi.org/10.1007/s11269-015-1178-5

Varela-Ortega, C., Blanco-Gutiérrez, I., Esteve, P., Bharwani, S., Fronzek, S., & Downing, T. E. (2016). How can irrigated agriculture adapt to climate change? Insights from the Guadiana Basin in Spain. Regional Environmental Change, 16(1), 59–70. https://doi.org/10.1007/s10113-014-0720-y

Syria

Cools, N., De Pauw, E., & Deckers, J. (2003). Towards an integration of conventional land evaluation methods and farmers' soil suitability assessment: a case study in north-western Syria. Agriculture, Ecosystems & Environment, 95(1), 327–342. https://doi.org/10.1016/S0167-8809(02)00045-2

Tunisia

Ferchichi, I., Marlet, S., & Zairi, A. (2017). How Farmers Deal with Water Scarcity in Community-Managed Irrigation SYSTEMS: A Case Study in Northern Tunisia. Irrigation and Drainage, 66(4), 556–566. https://doi.org/10.1002/ird.2135

Frija, A., Chebil, A., & Speelman, S. (2016). Farmers' Adaptation to Groundwater Shortage in the Dry Areas: Improving Appropriation or Enhancing Accommodation? Irrigation and Drainage, 65(5), 691–700. https://doi.org/10.1002/ird.1986

Gharsallaoui, M., Benincasa, C., Ayadi, M., Perri, E., Khlif, M., & Gabsi, S. (2011). Study on the impact of wastewater irrigation on the quality of oils obtained from olives harvested by hand and from the ground and extracted at different times after the harvesting. Scientia Horticulturae, 128(1), 23–29. https://doi.org/10.1016/j.scienta.2010.12.015

Khlifi, S., Ameur, M., Mtimet, N., Ghazouani, N., & Belhadj, N. (2010). Impacts of small hill dams on agricultural development of hilly land in the Jendouba region of northwestern Tunisia. Agricultural Water Management, 97(1), 50–56. ttps://doi.org/10.1016/j.agwat.2009.08.010

Latiri, K., Lhomme, J. P., Annabi, M., & Setter, T. L. (2010). Wheat production in Tunisia: Progress, inter-annual variability and relation to rainfall. European Journal of Agronomy, 33(1), 33–42. https://doi.org/10.1016/j.eja.2010.02.004

Mekki, I., Ghazouani, W., Closas, A., & Molle, F. (2017). Perceptions of groundwater degradation and mitigation responses in the Haouaria region in Tunisia. Groundwater for Sustainable Development, 5, 101–110. https://doi.org/10.1016/j.gsd.2017.05.001

Mougou, R., Mansour, M., Iglesias, A., Chebbi, R. Z., & Battaglini, A. (2011). Climate change and agricultural vulnerability: a case study of rain-fed wheat in Kairouan, Central Tunisia. Regional Environmental Change, 11(1), 137-142.

Poussin, J. C., Imache, A., Beji, R., Le Grusse, P., & Benmihoub, A. (2008). Exploring regional irrigation water demand using typologies of farms and production units: An example from Tunisia. Agricultural Water Management, 95(8), 973–983. https://doi.org/10.1016/j.agwat.2008.04.001

Turkey

Boz, I. (2016). Effects of environmentally friendly agricultural land protection programs: Evidence from the Lake Seyfe area of Turkey. Journal of Integrative Agriculture, 15(8), 1903–1914. https://doi.org/10.1016/S2095-3119(15)61271-0

Cakmak, B., Kibaroglu, A., Kendirli, B., & Gokalp, Z. (2008). Assessment of the irrigation performance of transferred schemes in Turkey: a case study analysis. Irrigation and Drainage, n/a-n/a. https://doi.org/10.1002/ird.452

Codron, J.-M., Adanacioğlu, H., Aubert, M., Bouhsina, Z., El Mekki, A. A., Rousset, S., Tonzanli, S., Yercan, M. (2014). The role of market forces and food safety institutions in the adoption of sustainable farming practices: The case of the fresh tomato export sector in Morocco and Turkey. Food Policy, 49, 268–280. https://doi.org/10.1016/j.foodpol.2014.09.006

Harris, L. M. (2009). Contested sustainabilities: assessing narratives of environmental change in southeastern Turkey. Local Environment, 14(8), 699–720. https://doi.org/10.1080/13549830903096452

Ilker, S., Kaplan, M., & Sahriye, S. (2007). Investigation of seasonal changes in nitrate contents of soils and irrigation waters in greenhouses located in Antalya-Demre region. Asian Journal Of Chemistry, 19(7), 5639–5646.

Lelandais, G. E. (2016). Drought, Social Inequalities, Adaptation, and Farmers’ Mobility in the Konya Plain of Turkey. In R. McLeman, J. Schade, & T. Faist (Eds.), Environmental Migration and Social Inequality (pp. 91–102). Springer, Cham. https:// doi.org/10.1007/978-3-319-25796-9_6

Miyata, S., & Fujii, T. (2007). Examining the socioeconomic impacts of irrigation in the Southeast Anatolia Region of Turkey. Agricultural Water Management, 88(1–3), 247–252. https://doi.org/10.1016/j.agwat.2006.11.001

Özerol, G., & Bressers, H. (2017). How do Farmers Align with the Agri-Environmental Changes in Irrigated Agriculture? A Case Study from the Harran Plain, Turkey. Irrigation and Drainage, 66(1), 45–59. https://doi.org/10.1002/ird.2064

Yaslioglu, E., Akkaya Aslan, S. T., Kirmikil, M., Gundogdu, K. S., & Arici, I. (2009). Changes in Farm Management and Agricultural Activities and Their Effect on Farmers’ Satisfaction from Land Consolidation: The Case of Bursa–Karacabey, Turkey. European Planning Studies, 17(2), 327–340. https://doi.org/10.1080/09654310802553639
