# Supplementary material for: Adaptations in irrigated agriculture in the Mediterranean region: an overview and spatial analysis of implemented strategies
Source: Reg Environ Change. 2019 Apr 24;19(5):1401–16. doi: 10.1007/s10113-019-01494-8 (PMC6531414; doi:10.1007/s10113-019-01494-8)

Farm level adaptations in irrigated agriculture in the Mediterranean region:
An overview and spatial analysis of implemented strategies
Regional Environmental Change | Kina S. Harmanny & Žiga Malek; kinaharmanny@outlook.com

Online Resource 2 High resolution images of spatial context maps

All adaptations

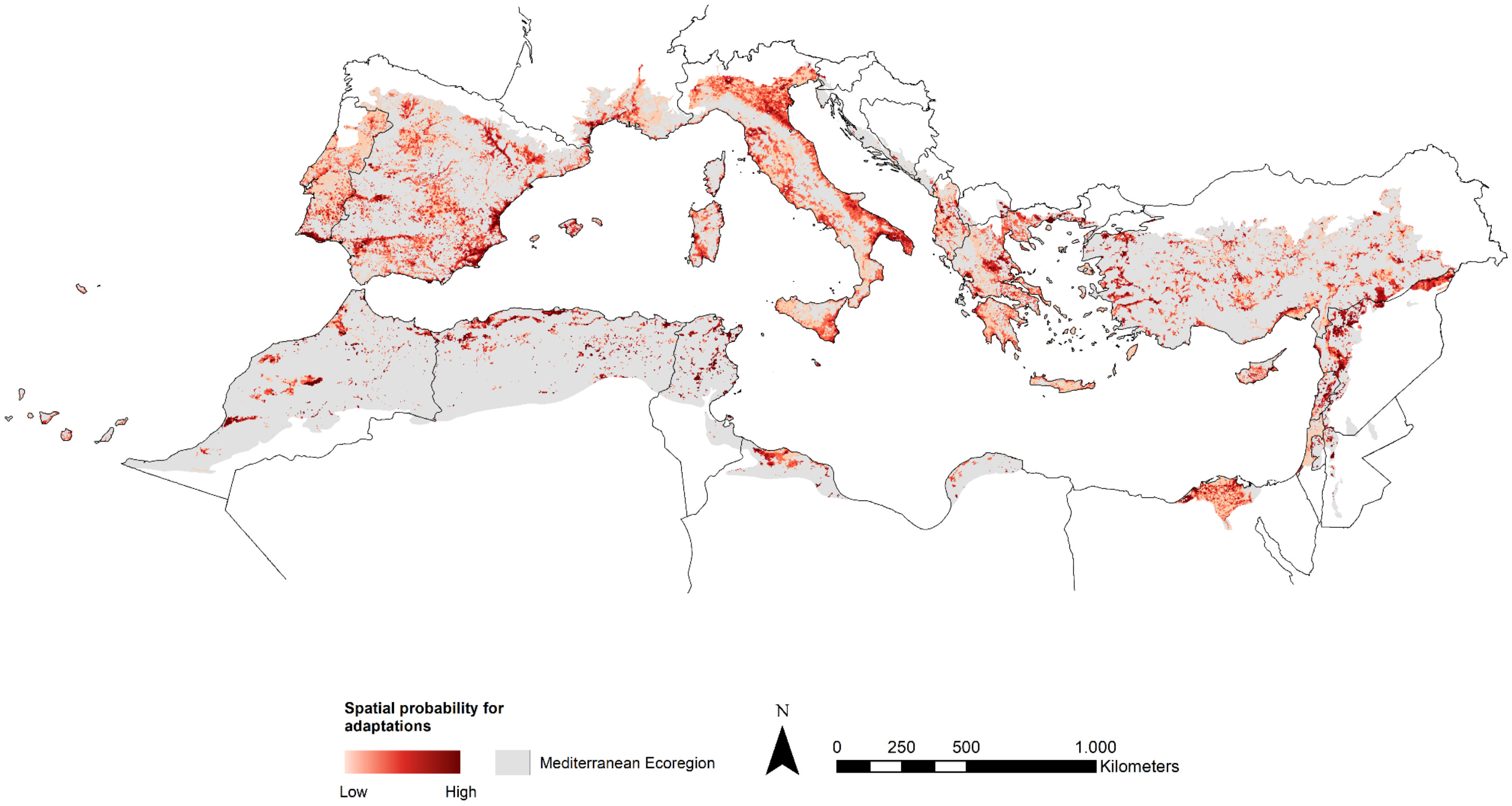

Water management

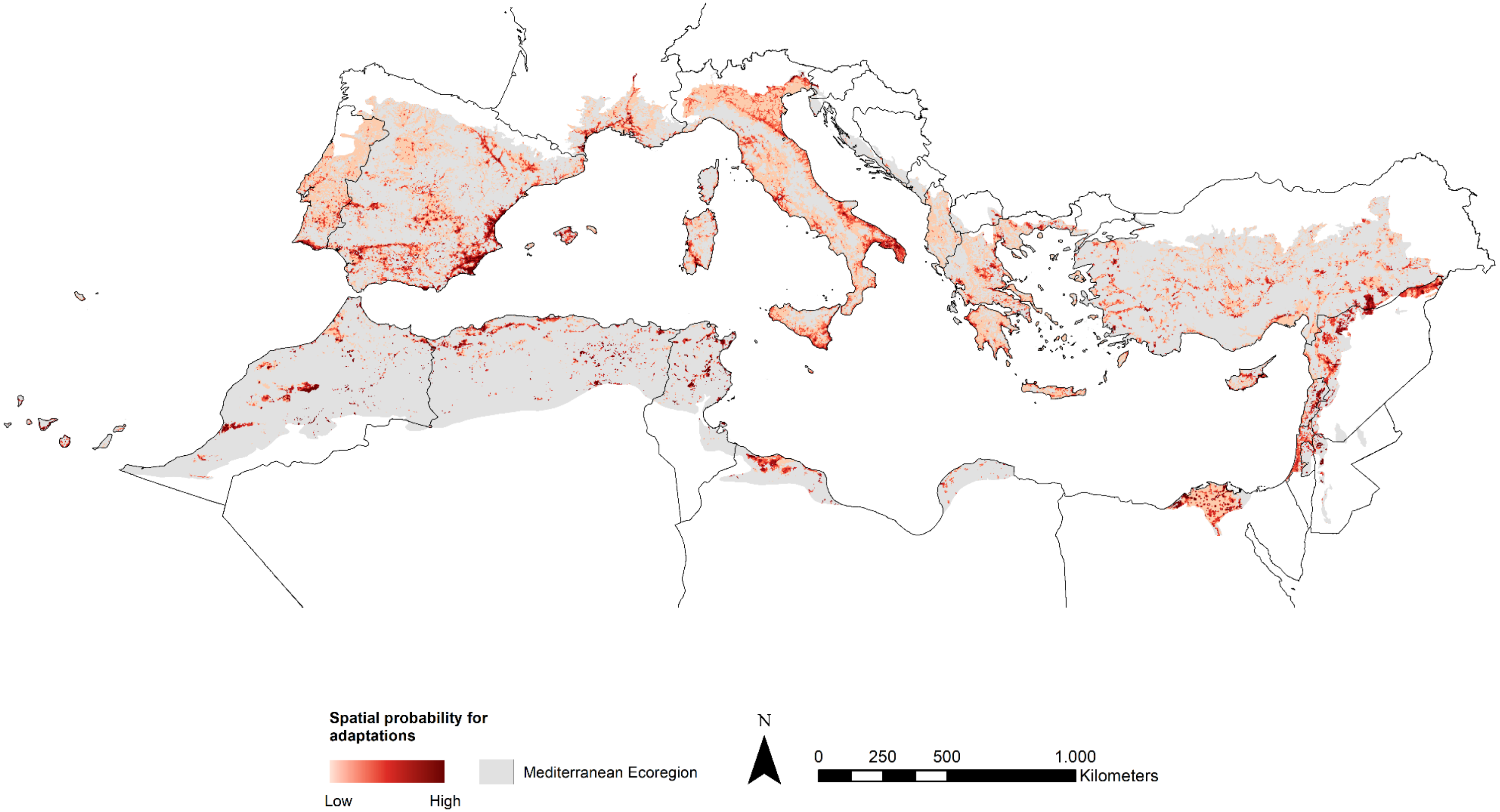

Farm level adaptations in irrigated agriculture in the Mediterranean region:
An overview and spatial analysis of implemented strategies
Regional Environmental Change | Kina S. Harmanny & Žiga Malek; kinaharmanny@outlook.com

Sustainable resource management

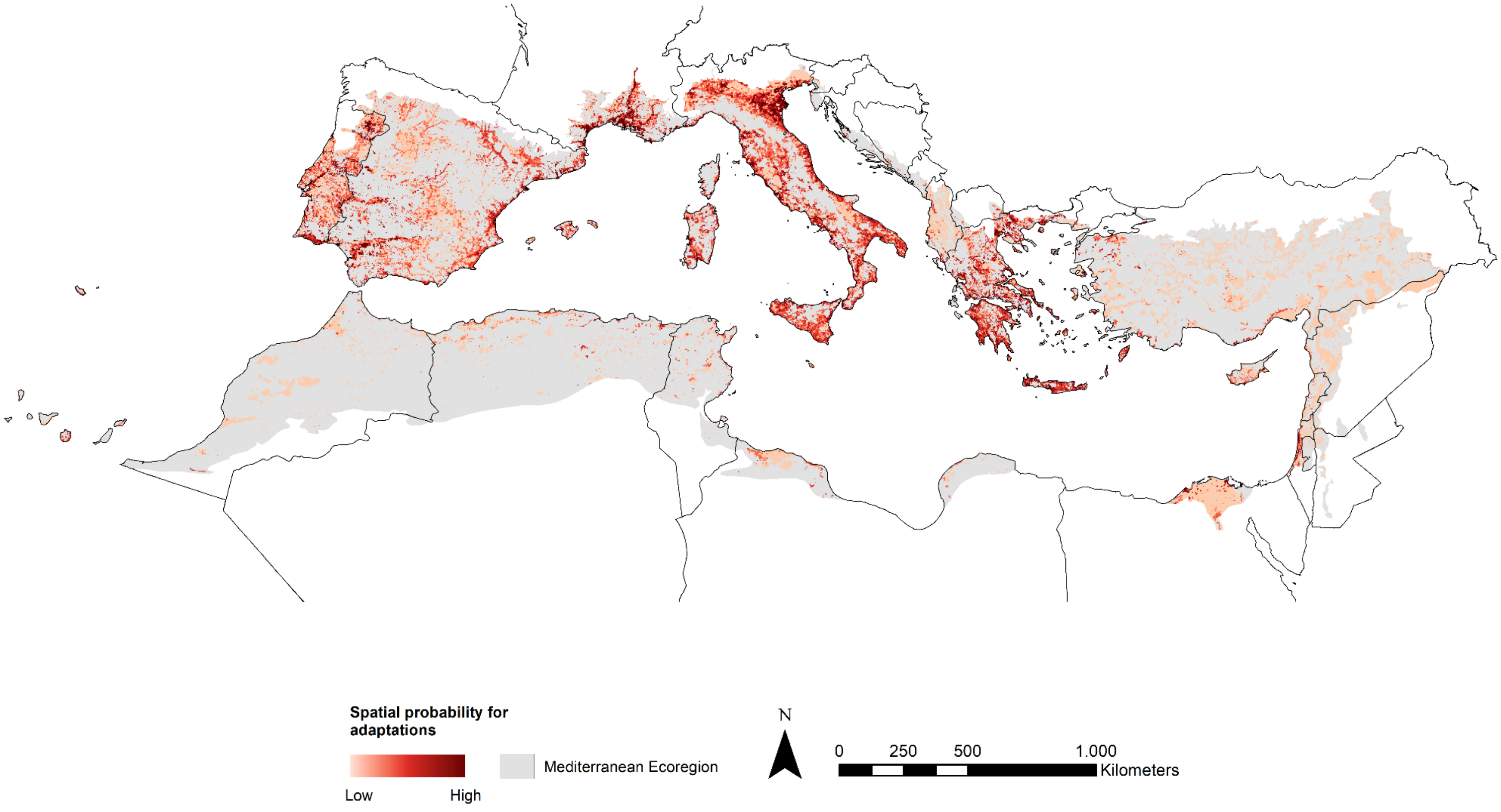

Farm level adaptations in irrigated agriculture in the Mediterranean region:
An overview and spatial analysis of implemented strategies
Regional Environmental Change | Kina S. Harmanny & Žiga Malek; kinaharmanny@outlook.com

Technological developments

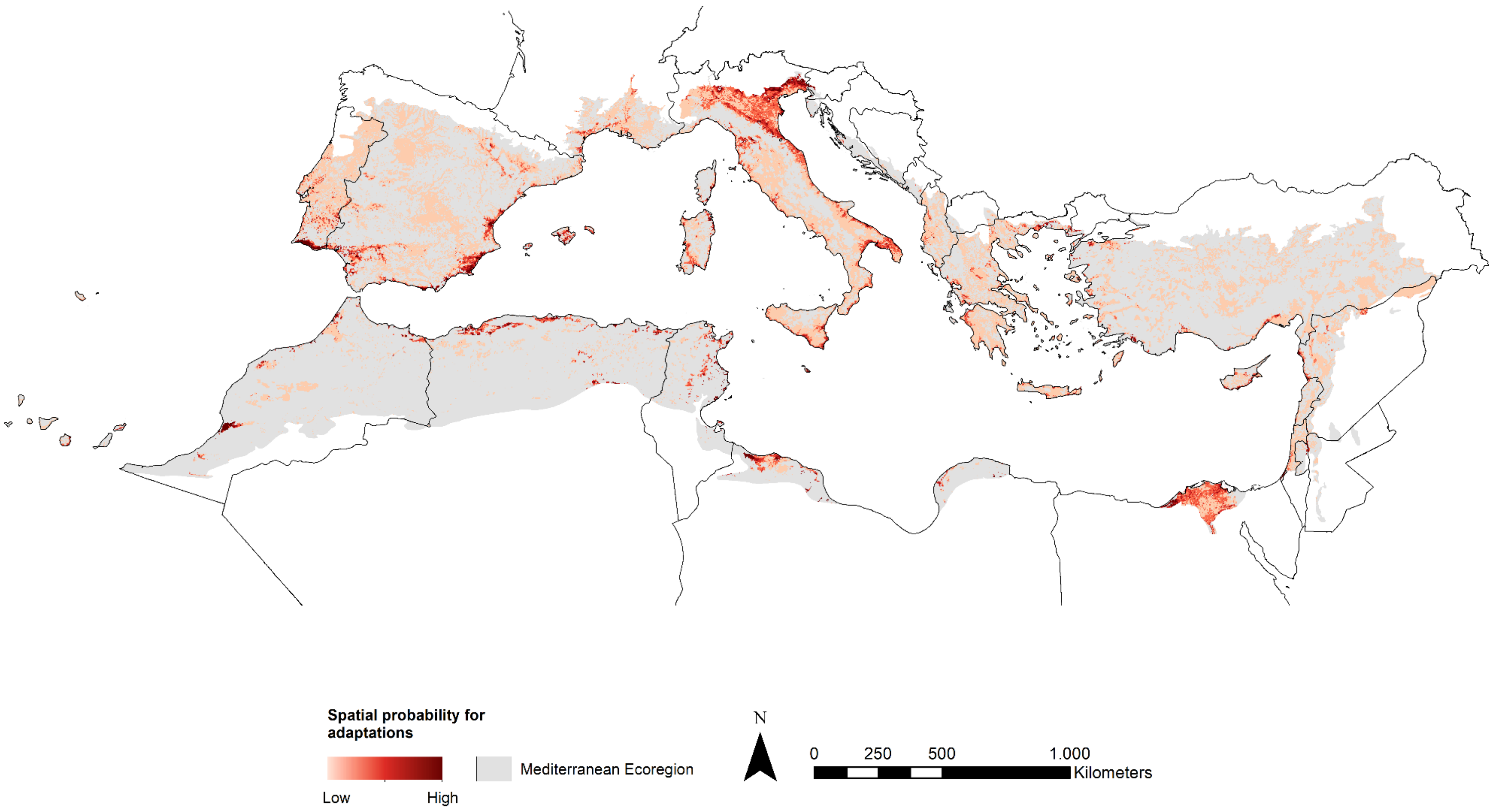

Farm production practices

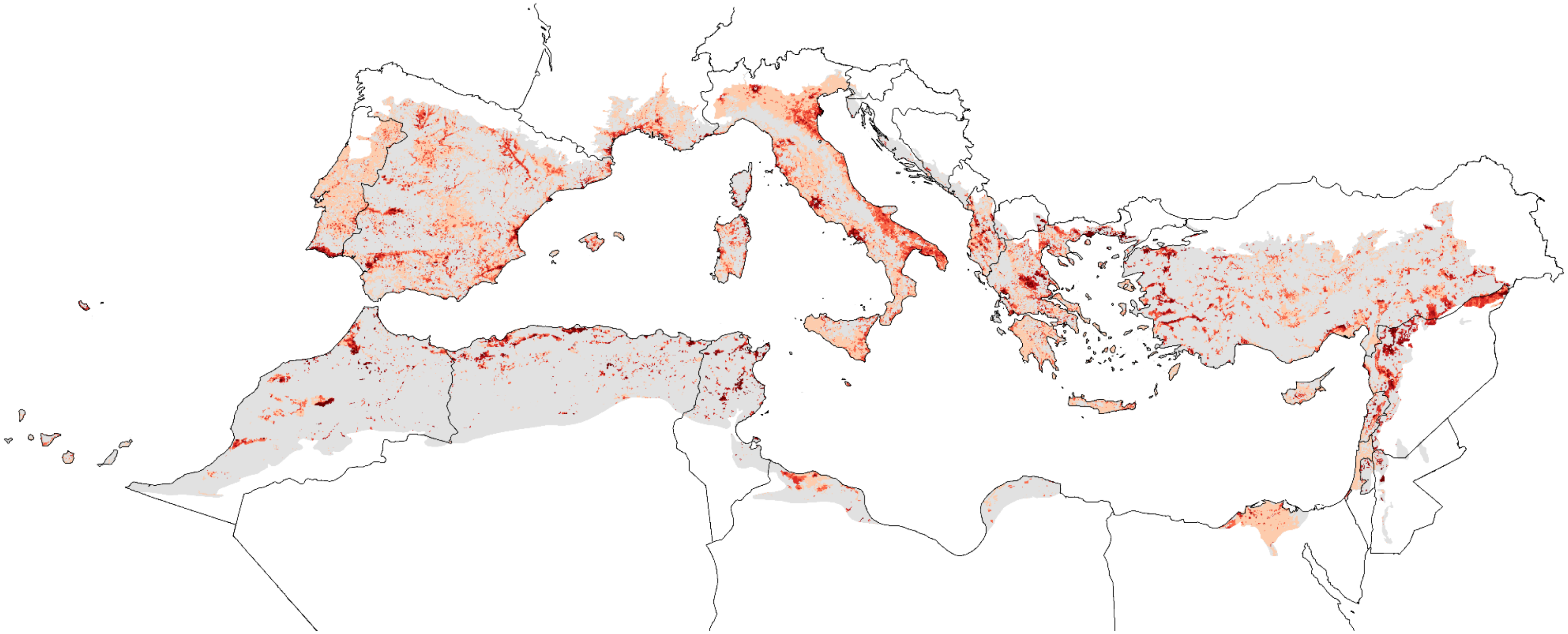

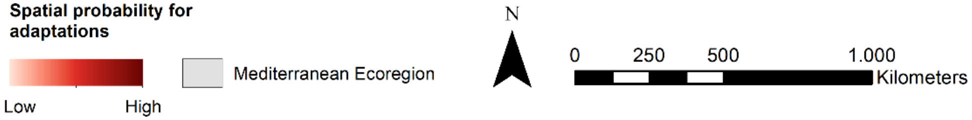

Farm level adaptations in irrigated agriculture in the Mediterranean region:
An overview and spatial analysis of implemented strategies
Regional Environmental Change | Kina S. Harmanny & Žiga Malek; kinaharmanny@outlook.com

Farm management

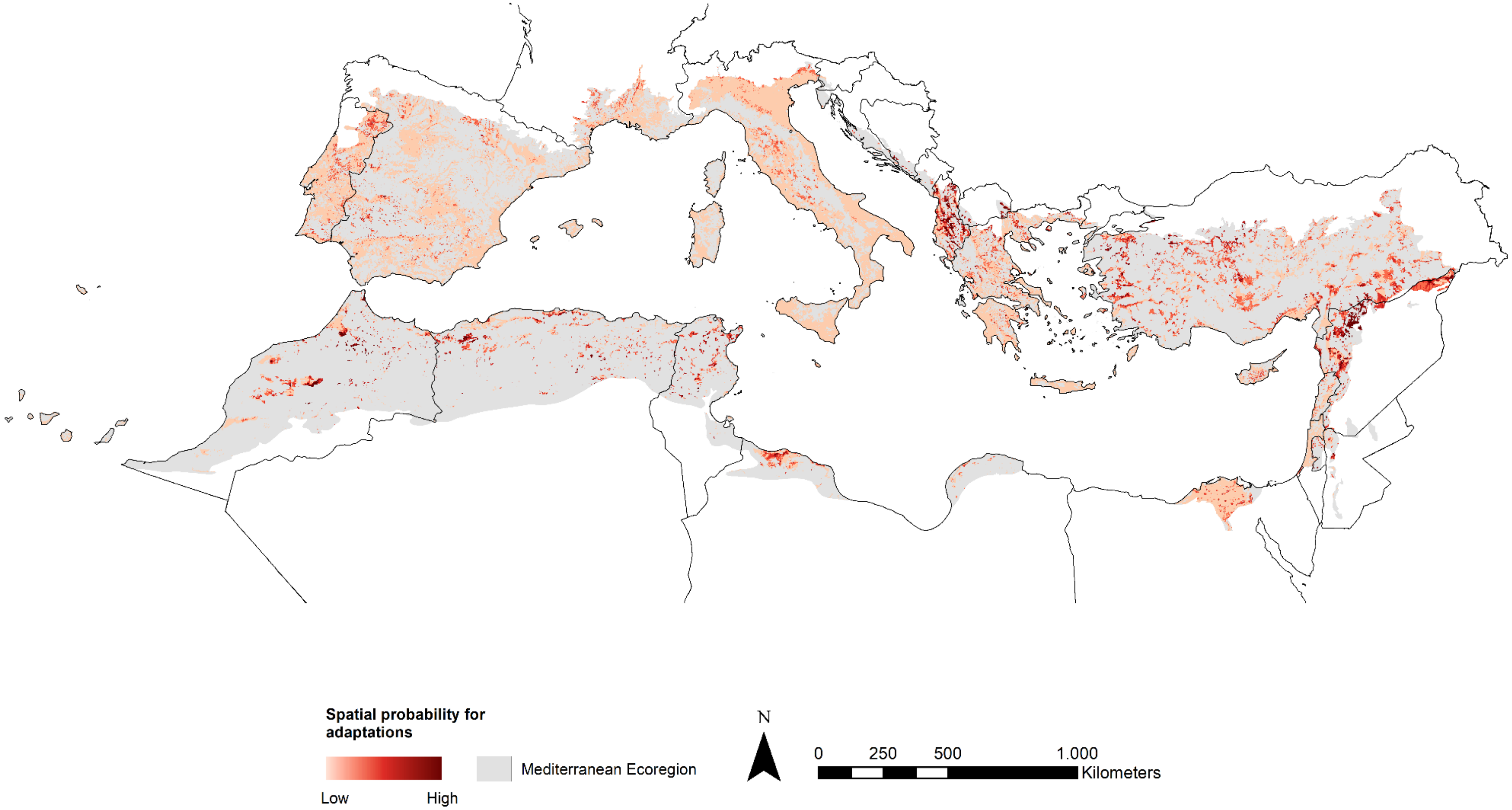

Supplement: Supplementary file 2 — (PDF 1956 kb) [file 10113_2019_1494_MOESM2_ESM.pdf]
