# Supplementary material for: Adaptations in irrigated agriculture in the Mediterranean region: an overview and spatial analysis of implemented strategies
Source: Reg Environ Change. 2019 Apr 24;19(5):1401–16. doi: 10.1007/s10113-019-01494-8 (PMC6531414; doi:10.1007/s10113-019-01494-8)
Supplement: Supplementary file 3 — (PDF 152 kb) [file 10113_2019_1494_MOESM3_ESM.pdf]

Online Resource 3 Correlation between the included variables

Variables	Altitude	CEC (soil)	Clay (soil)	Drainage (soil)	Solar radiation	PET	pH (soil)	Population density	Precipitation	Road distance	Rural population	Sand (soil)	Slope	soil depth	Market influence	Temperature	Organic content (soil)
Market accessibility	-0.331	0.098	0.202	0.053	-0.037	0.029	-0.036	0.227	0.088	-0.276	0.152	-0.145	-0.105	0.152	0.730	0.176	-0.027
Altitude		0.041	-0.363	-0.006	-0.078	-0.096	0.081	-0.177	-0.067	0.078	-0.188	0.282	0.339	-0.338	-0.311	-0.645	0.097
CEC (soil)			0.231	-0.024	-0.039	-0.025	0.141	0.016	0.113	-0.052	0.005	-0.168	0.104	-0.055	0.047	-0.015	0.066
Clay (soil)				-0.094	0.064	0.162	0.044	0.118	0.107	-0.063	0.216	-0.788	-0.352	0.309	0.071	0.274	0.073
Drainage (soil)					-0.006	-0.048	0.261	-0.050	0.024	-0.001	-0.081	0.098	-0.041	0.034	0.061	-0.007	-0.014
Solar radiation						0.787	0.354	0.159	-0.645	0.074	0.270	0.031	-0.176	0.121	-0.438	0.716	-0.148
PET							0.327	0.157	-0.582	0.089	0.309	-0.057	-0.279	0.252	-0.388	0.647	-0.191
pH (soil)								0.032	-0.458	0.031	0.112	-0.072	-0.188	0.110	-0.156	0.187	-0.201
Population dens.									-0.121	-0.082	0.191	-0.089	-0.099	0.107	0.063	0.207	-0.046
Precipitation										-0.087	-0.243	-0.158	0.306	-0.156	0.293	-0.339	0.281
Road distance											-0.012	0.053	-0.002	-0.014	-0.275	0.004	0.003
Rural population												-0.123	-0.123	0.107	-0.074	0.282	-0.042
Sand (soil)													0.332	-0.177	-0.093	-0.155	-0.176
Slope														-0.528	-0.016	-0.264	0.204
Soil depth															0.019	0.264	-0.462
Market influence																-0.116	0.037
Temperature																	-0.154
Organic content (soil)																	
