# Supplementary material for: Adaptations in irrigated agriculture in the Mediterranean region: an overview and spatial analysis of implemented strategies
Source: Reg Environ Change. 2019 Apr 24;19(5):1401–16. doi: 10.1007/s10113-019-01494-8 (PMC6531414; doi:10.1007/s10113-019-01494-8)
Supplement: Supplementary file 4 — (PDF 146 kb) [file 10113_2019_1494_MOESM4_ESM.pdf]

Online Resource 4 Spatial autocorrelation for the identified locations

Spatial autocorrelation for the identified locations. Moran's I statistic presents the measure for spatial autocorrelation and ranges between -1 (completely dispersed/negative autocorrelation), to 1 (completely clustered/high spatial autocorrelation). Values closer to 0 indicate no autocorrelation. The z-score shows the statistical significance of the single variable on the spatial autocorrelation. Significance values for variables: *p<0.05, **p<0.01, ***p<0.001

Variable	Moran's I	z-score	Spatial pattern type
Market accessibility	0.96***	7.92	Clustered
Altitude	0.77***	6.56	Clustered
CEC (soil)	0.17	1.49	No autocorrelation
Clay (soil)	0.16	1.37	No autocorrelation
Drainage (soil)	0.3**	2.67	Slightly clustered
Solar radiation	0.02	0.69	No autocorrelation
PET	0.76	6.29	Clustered
pH (soil)	0.42***	3.51	Slightly clustered
Population density	0.05	0.53	No autocorrelation
Precipitation	0.38**	3.24	Slightly clustered
Road distance	0.25*	2.33	Slightly clustered
Rural population	0.07	0.78	No autocorrelation
Sand (soil)	0.24*	2.04	Slightly clustered
Slope	0.45***	3.98	Slightly clustered
Soil depth	0.29*	2.53	Slightly clustered
Market influence	0.65***	5.46	Clustered
Temperature	0.47***	3.92	Slightly clustered
Organic content (soil)	0.05	0.52	No autocorrelation
